# UvATG6 Interacts with BAX Inhibitor 1 Proteins and Plays Critical Roles in Growth, Conidiation, and Virulence in *Ustilaginoidea virens*

**DOI:** 10.1128/spectrum.04898-22

**Published:** 2023-04-27

**Authors:** Lifan Gu, Yufu Wang, Songlin Xie, Yueran Liu, Jiali Yan, Weixiao Yin, Chaoxi Luo

**Affiliations:** a Hubei Key Laboratory of Plant Pathology, College of Plant Science and Technology, Huazhong Agricultural University, Wuhan, China; Agroscope

**Keywords:** ATG6, autophagy, Bax inhibitor 1, *Ustilaginoidea virens*, virulence

## Abstract

Autophagy and apoptosis are evolutionarily conserved catabolic processes involved in regulating development and cellular homeostasis. Bax inhibitor 1 (BI-1) and autophagy protein 6 (ATG6) perform essential functions in these roles, such as cellular differentiation and virulence in various filamentous fungi. However, the functions of ATG6 and BI-1 proteins in development and virulence in the rice false smut fungus Ustilaginoidea virens are still poorly understood. In this study, UvATG6 was characterized in U. virens. The deletion of *UvATG6* almost abolished autophagy in *U. virens* and reduced growth, conidial production and germination, and virulence. Stress tolerance assays showed that *UvATG6* mutants were sensitive to hyperosmotic, salt, and cell wall integrity stresses but were insensitive to oxidative stress. Furthermore, we found that UvATG6 interacted with UvBI-1 or UvBI-1b and suppressed Bax-induced cell death. We previously found that UvBI-1 could suppress Bax-induced cell death and was a negative regulator of mycelial growth and conidiation. Unlike UvBI-1, UvBI-1b could not suppress cell death. *UvBI-1b*-deleted mutants exhibited decreased growth and conidiation, while the *UvBI-1* and *UvBI-1b* double deletion reduced the phenotype, indicating that *UvBI-1* and *UvBI-1b* antagonistically regulate mycelial growth and conidiation. In addition, the *UvBI-1b* and double mutants exhibited decreased virulence. Our results provide evidence of the cross talk of autophagy and apoptosis in *U. virens* and give clues for studying other phytopathogenic fungi.

**IMPORTANCE** *Ustilaginoidea virens* causes destructive panicle disease in rice, significantly threatening agricultural production. UvATG6 is required for autophagy and contributes to growth, conidiation, and virulence in *U. virens*. Additionally, it interacts with the Bax inhibitor 1 proteins UvBI-1 and UvBI-1b. UvBI-1 suppresses cell death induced by Bax, unlike UvBI-1b. *UvBI-1* negatively regulates growth and conidiation, while *UvBI-1b* is required for these phenotypes. These results indicate that UvBI-1 and UvBI-1b may antagonistically regulate growth and conidiation. In addition, both of them contribute to virulence. Additionally, our results suggest cross talk between autophagy and apoptosis, contributing to the development, adaptability, and virulence of *U. virens*.

## INTRODUCTION

Autophagy is a degradative process by which double-membrane vesicles (autophagic vesicles or autophagosomes) engulf cytoplasmic organelles and proteins and then fuse with lysosomes in which the cytoplasmic material is hydrolyzed and recycled (1). Autophagy is a cytoprotective mechanism that prevents the death of cells by preventing them from undergoing apoptosis. Conversely, autophagic cell death is characterized by the massive accumulation of autophagic vacuoles in the cytoplasm of cells (2).

In most cases, autophagy and apoptosis can inhibit each other (3, 4), but both can kill cells, indicating that their regulation is coordinated. Molecular cross talk between these two processes has been described. Some regulators, including apoptosis p53 and Beclin 1 (ortholog of yeast Atg6), have been identified to control apoptosis and autophagy (1). Caspases with foundational roles in apoptotic cell death can cleave Beclin 1, thereby destroying its proautophagic activity (5, 6). In addition, the interacting proteins of Atg6, including the antiapoptotic protein Bcl-2, were identified to play connecting roles between apoptosis and autophagy (7, 8), indicating that Atg6 plays an essential role in the cross talk between apoptosis and autophagy.

The phosphatidylinositol 3-kinase (PI3K) complex, including Beclin 1, is required for the first step of autophagosome formation (9, 10). In addition, as part of the PI3K complex, Beclin 1 has been identified to interact with Bcl-2 (as well as its homolog Bcl-X_L_), which inhibits its autophagic function (7, 11, 12). The Bcl-2 protein family includes antiapoptotic proteins (Bcl-2 and Bcl-X_L_) and proapoptotic proteins (Bad, Bax, and Bak) (11, 13). Furthermore, Bcl-2 and Bcl-X_L_ can interact with Bax to neutralize its proapoptotic function (14). In addition, the Bax inhibitor 1 (BI-1) protein was found to suppress apoptosis induced by Bax (15). Although there is no direct interaction between BI-1 and Bax, BI-1 can interact with Bcl-2 and Bcl-X_L_ to regulate endoplasmic reticulum (ER) calcium homeostasis, enhancing apoptosis suppression (15–19). In addition, BI-1 also plays a role in early adaptive responses against ER stress by interacting with IRE1α and inhibiting its mediated unfolded protein response (UPR) (20). In plants, BI-1 interacts with ATG6 to regulate autophagy, which is required for both prosurvival and prodeath functions (21).

Ustilaginoidea virens, the agent of rice false smut, causes significant yield losses annually and has become one of the major fungal diseases of rice. Additionally, U. virens produces mycotoxins that harm animals and plants (22, 23). Several autophagy-related genes (ATGs) in *U. virens* have been characterized. The *UvATG8*, *UvATG7*, and *UvATG14* genes have been identified to contribute to growth, conidiation, and virulence (24–26). In addition, UvATG14 interacts with UvATG6 and MoATG6 from Magnaporthe oryzae but not with ScATG6 from Saccharomyces cerevisiae (26). Although these genes have been studied, the other ATGs in *U. virens* still need to be explored.

In this study, we found that UvATG6 was required for autophagy and contributed to growth, conidiation, and virulence. In addition, UvATG6 interacted with UvBI-1 and UvBI-1b, and both of them may antagonistically regulate growth and conidiation in *U. virens*. These results highlight that the BI-1 and UvATG6 proteins play essential roles in development, stress tolerance, and virulence and provide evidence of cross talk between autophagy and apoptosis in *U. virens*.

## RESULTS

### Identification and characterization of UvATG6 in *U. virens*.

**FIG 1:**
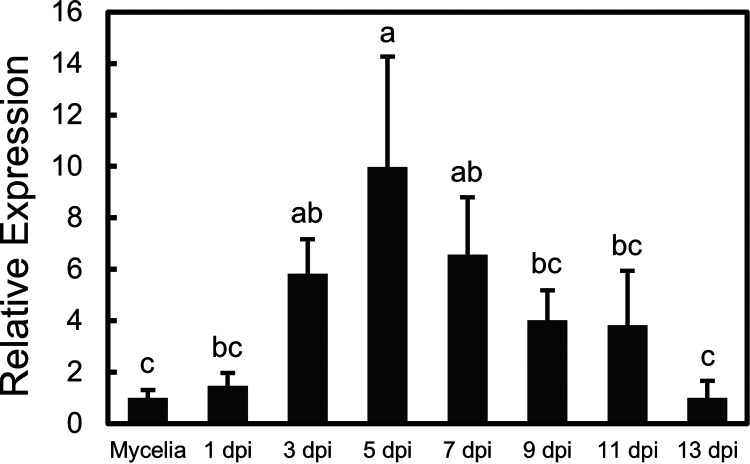
Expression levels of *UvATG6* at infection stages. The expression of UvATG6 at the indicated stages was detected using RT-qPCR. A susceptible rice cultivar (Wanxian-98) was inoculated with *U. virens* strain JS60-2. Mycelia were cultured for 7 days, and the panicles at 1, 3, 5, 7, 9, 11, and 13 dpi were collected. RNA was extracted. A fragment of the β-tubulin gene from *U. virens* was used as an internal reference, and the expression level of mycelia was normalized to a value of 1. The error bars represent the standard deviations derived from three replicates. The letters represent statistical significance (*P < *0.05).

To investigate the function of ATG proteins in *U. virens*, we selected the ATG6 protein (UV8b_02704 in GenBank, https://www.ncbi.nlm.nih.gov/protein/2083616542) for further research. *UvATG6* is predicted to encode a 495-amino-acid (aa) protein in *U. virens* strain Uv-8b and contains a predicted autophagy (APG) domain (see Fig. S1A in the supplemental material). We amplified *UvbATG6* from the cDNA of strain JS60-2, and the sequence is the same except for two sites (Fig. S1B). The differences in the sequences are probably because they are from different strains. Sequence alignment and phylogenetic analysis showed that ATG6 was conserved in fungi and plants (Fig. S2). We also detected gene transcription in JS60-2 at different stages, and *UvATG6* exhibited higher transcriptional levels at 3 to 7 days postinoculation (dpi) (Fig. 1).

To investigate the functions of *UvATG6*, we obtained deletion mutants and complemented strains using methods described previously (27). These strains were verified using different primers (Fig. S3A to D), and reverse transcription-PCR (RT-PCR) was used to evaluate *UvATG6* expression (Fig. S3E).

### UvATG6 is indispensable for autophagy.

**FIG 2:**
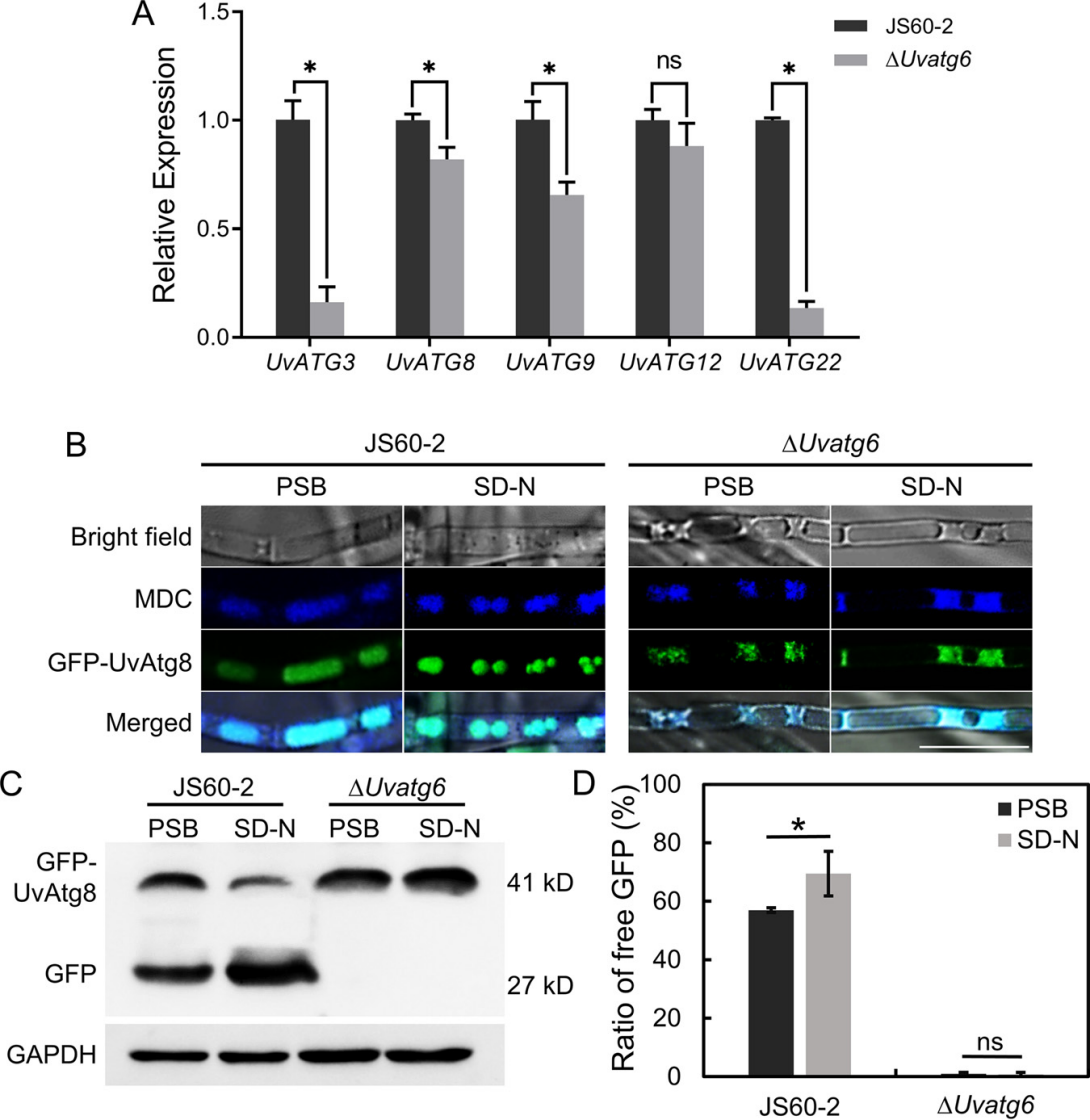
UvATG6 is essential for autophagy. (A) Expression of autophagy-related genes in *Uvatg6*-deleted transformants. The indicated genes were detected by RT-qPCR. The β-tubulin gene was used as an internal reference, and the expression level in wild-type strain J60-2 was normalized to a value of 1. The error bars represent the standard deviations, and the asterisks represent significant differences (*P < *0.05). ns, not significant. (B) Autophagy fluorescence observation of *Uvatg6*-deleted transformants. GFP-UvATG8 was expressed in JS60-2 and *Uvatg6* mutant strains. The strains were cultured in PSB for 3 days and treated in medium (SD−N) for 6 h. The mycelia were collected and stained with MDC for fluorescence observation. Bar = 10 μm. (C and D) Detection of GFP-UvAtg8 degradation. Mycelial protein was extracted and detected by anti-GFP and anti-GAPDH antibodies. The protein bands were analyzed using ImageJ. The experiments were performed in three independent biological replicates with three technical replicates each time. The error bar represents the standard deviation, and the asterisk represents a significant difference (*P < *0.05).

To monitor the autophagy level in UvATG6 mutants, the expression levels of several autophagy-related genes, including *UvATG3*, *UvATG8*, *UvATG9*, *UvATG12*, and *UvATG22*, were determined. Compared to wild-type strain JS60-2, the levels of these genes were decreased in the mutants, except for *UvATG12*, which exhibited no significant difference (Fig. 2A). ATG8 is a widely used marker for nonspecific autophagy, and green fluorescent protein (GFP)-fused ATG8 is used to monitor autophagic processes. GFP is cleaved from GFP-ATG8 when this process is normal. We transformed GFP-UvATG8 into wild-type strain JS60-2 and *UvATG6* mutants, and the transformants were verified using different primer pairs (Fig. S4). After starvation treatment, stronger fluorescence was observed throughout the monodansylcadaverine (MDC)-stained vacuoles in the wild-type strains, indicating that autophagy was induced. Compared to wild-type strain JS60-2, low fluorescence intensity was observed in the vacuoles of *UvATG6* mutants with or without starvation treatment (Fig. 2B). We also determined the autophagy level using Western blotting. The autophagy level was estimated by calculating free GFP compared with the total GFP-UvATG8. The results showed that the autophagy level in JS60-2 was increased after starvation treatment, indicating that starvation promotes autophagy. In addition, the autophagy level in the *UvATG6* mutants was abolished because free GFP was almost unobservable, irrespective of starvation treatment (Fig. 2C and D). These results suggest that *UvATG6* is essential for autophagy in *U. virens*.

### *UvATG6* is essential for mycelial growth and conidiation.

**FIG 3:**
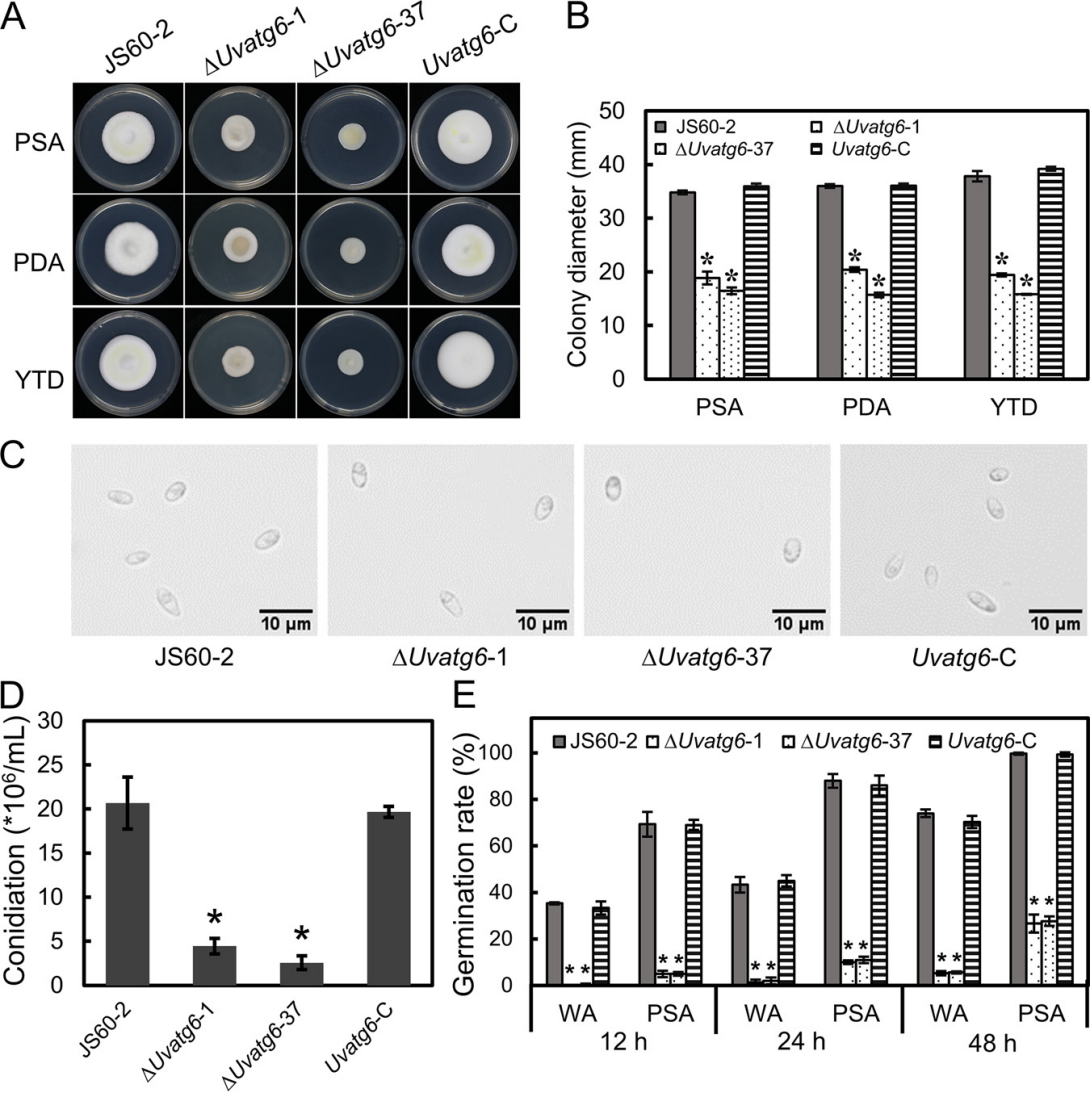
Mycelial growth and conidiation of Δ*Uvatg6* transformants. (A) Colonial morphology of Δ*Uvatg6* transformants on PSA, PDA, and YTD. (B) Statistical analysis of growth. (C) Conidial morphology of Δ*Uvatg6* transformants. (D) Statistical analysis of conidiation. (E) Analysis of the conidial germination rate. The experiments were performed in three independent biological replicates with three technical replicates each time. Error bars represent the standard deviations, and asterisks represent significant differences (*P < *0.05).

The *UvATG6* mutants were cultured on potato sucrose agar (PSA), potato dextrose agar (PDA), and yeast extract-tryptone-dextrose (YTD) plates to investigate whether *UvATG6* contributes to growth. The mutants exhibited decreased growth compared to wild-type strain JS60-2, and the aerial mycelia of the mutants were also decreased, indicating that *UvATG6* is required for vegetative growth (Fig. 3A and B). In addition, the conidiation and germination rates were also investigated. The morphology of the conidia exhibited no difference (Fig. 3C). Still, the conidiation of the *UvATG6* mutants was decreased (Fig. 3D). Additionally, the germination rate of the mutants was decreased significantly on water agar (WA) and PDA plates (Fig. 3E and Fig. S5). All of these results suggest that *UvATG6* is essential for vegetative growth and conidiation.

### UvATG6 is involved in stress tolerance.

We investigated the tolerance of *UvATG6* mutants to different stressors, including sorbitol, NaCl, sodium dodecyl sulfate (SDS), congo red (CR), calcofluor white (CFW), and H_2_O_2_. These results showed that the UvATG6 mutants were sensitive to hyperosmotic conditions, salt, and cell wall integrity stressors but were insensitive to oxidative stress (Fig. S6). These results suggest that *UvATG6* is involved in stress tolerance.

### Deletion of UvATG6 causes a loss of the virulence of *U. virens*.

**FIG 4:**
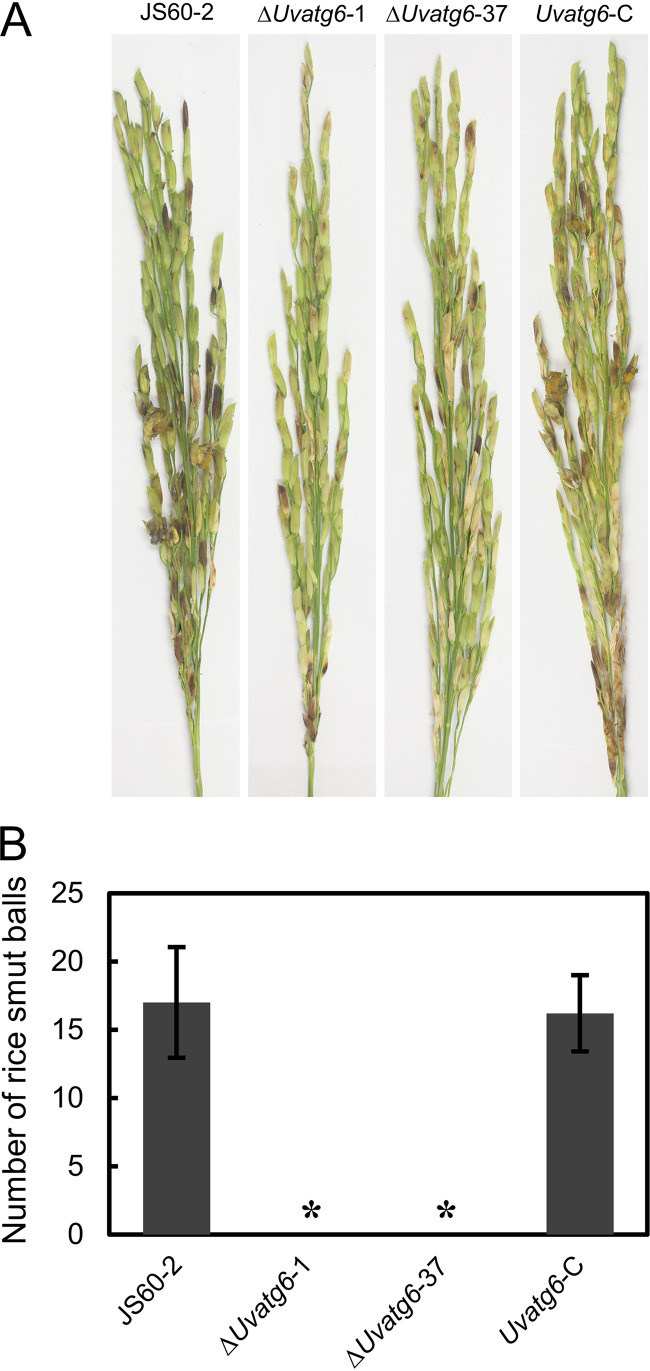
Virulence assay of Δ*Uvatg6* transformants. (A) Disease symptom observation of Δ*Uvatg6* transformants. (B) Statistical analysis of the number of smut balls. The number of smut balls in individual rice panicles was measured. At least three independent biological experiments were performed with at least 10 replicates in each test. Asterisks represent significant differences (*P < *0.05).

Virulence assays were performed in susceptible rice to examine whether UvATG6 contributes to the virulence of *U. virens*. A mixture suspension of mycelia and conidia was injected into the rice panicles. At 21 dpi, UvATG6 mutants produced a few smut balls in rice, in contrast to the typical and many false smut balls of the wild-type and complemented strains (Fig. 4). This result suggests that UvATG6 is essential for the virulence of *U. virens*.

### UvATG6 interacts with UvBI-1 and UvBI-1b.

Since plant BI-1 interacts with ATG6 to regulate autophagy (21), we tested whether UvATG6 can interact with the Bax inhibitor 1 protein in *U. virens*. In a previous study, we identified two Bax inhibitor 1 proteins in *U. virens*, UvBI-1 and UvBI-1b, and the function of UvBI-1 has been explored (27). UvBI-1b is a 349-amino-acid protein, and sequence analysis showed that UvBI-1b contained a BI-1-like domain, including six transmembrane regions (Fig. S7A). Furthermore, phylogenetic analysis revealed that UvBI-1 and UvBI-1b belonged to different groups and indicated that UvBI-1b was a conserved protein in fungi (Fig. S7B). The transcriptional patterns at various infection stages and the vegetative stage were investigated. The results showed that the expression levels were almost 350 times higher at 3 dpi and 200 times higher at 5 dpi (Fig. S7C). The distinct expression pattern of *UvBI-1* versus *UvBI-1b* may reflect the specific roles of these two members at different infection stages and indicates the potential functions of *UvBI-1b* during the infection stage (27).

**FIG 5:**
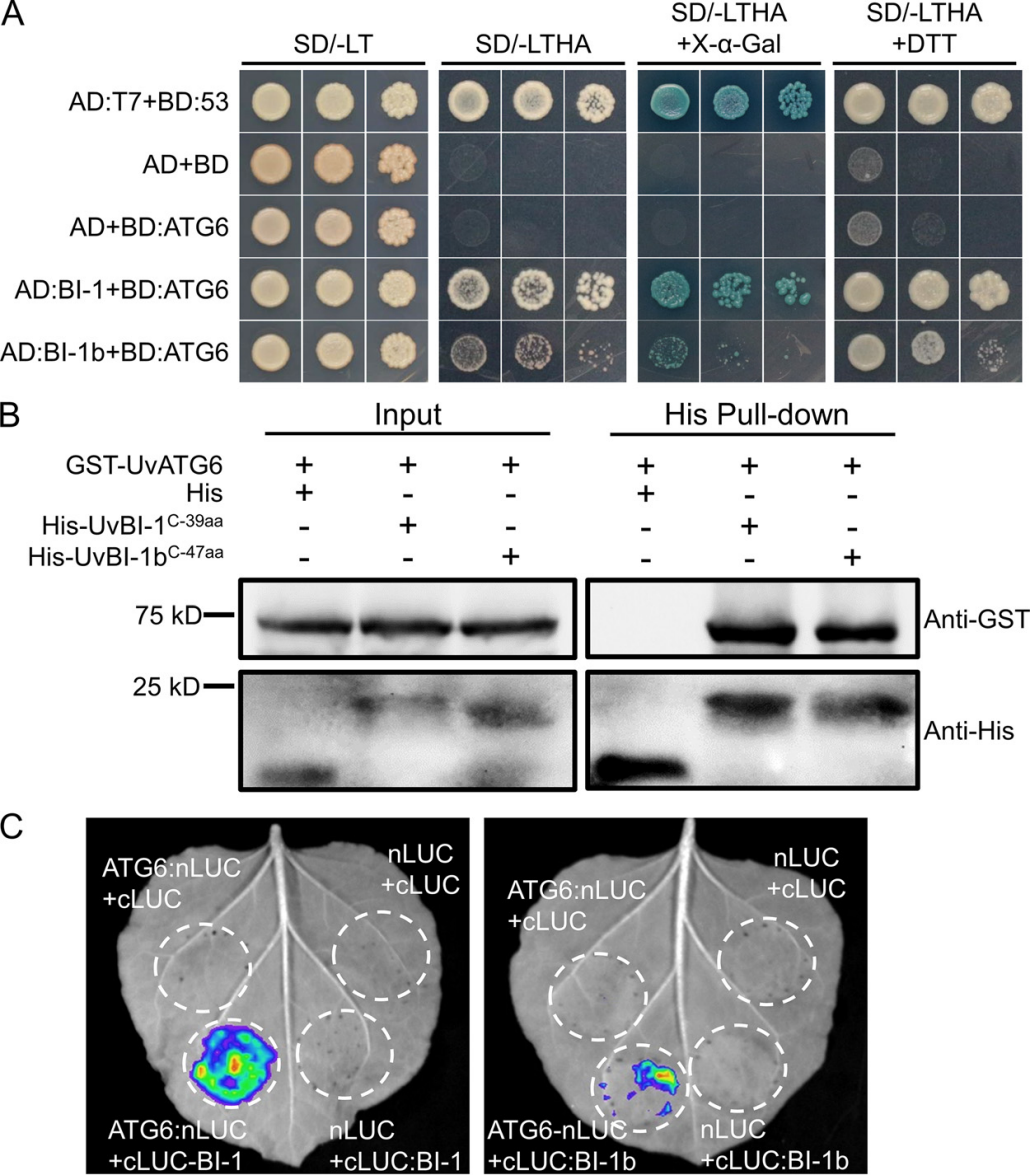
UvATG6 interacts with UvBI-1 and UvBI-1b. (A) Yeast two-hybrid assay for the interaction between UvATG6 and UvBI-1 or UvBI-1b. The plasmids pGADT7 (AD) and pGBKT7 (BD) were fused with the indicated genes and cotransformed into yeast strain AH109. The transformants were cultured on SD/−Leu−Trp (SD/-LT) and SD/−Leu−Trp−His−Ade (SD/-LTHA) or SD/−Leu−Trp−His−Ade with X-α-Gal (SD/-LTHA+X-α-Gal). AD:T7 and BD:53 were used as positive controls. (B) Pulldown assay for the interaction between UvATG6 and UvBI-1 or UvBI-1b. The C-terminal 39 aa of UvBI-1 (His-UvBI-1^C-39aa^) and 47 aa of UvBI-1b (His-UvBI-1b^C-47aa^) were expressed in E. coli. The recombinant protein His–UvBI-1^C-39aa^ or His–UvBI-1b^C-47aa^ or His immobilized on Ni-nitrilotriacetic acid (NTA) resin was incubated with the GST-ATG6 protein. The input and pulldown samples were detected by Western blotting with anti-GST and anti-His antibodies. (C) Luciferase complementation for the interaction between UvATG6 and UvBI-1 or UvBI-1b. The proteins were expressed in N. benthamiana leaves by Agrobacterium tumefaciens infiltration, and fluorescence was observed after 48 h.

BI-1 proteins contain transmembrane domains, and the C terminus is essential for interactions with ATG6. The C-terminal 39 aa of UvBI-1 and 47 aa of UvBI-1b were coexpressed in yeast with UvATG6. The yeast cells containing UvATG6 and UvBI-1^C-39aa^ or UvBI-1b^C-47aa^ grew on medium minus Trp, Leu, Ade, and His. Blue colonies were observed on plates containing 5-bromo-4-chloro-3-indolyl-d-galactoside (X-α-Gal). In addition, the yeast cells exhibited better growth on plates containing dithiothreitol (DTT), an ER stressor (Fig. 5A). UvBI-1^C-39aa^ and UvBI-1b^C-47aa^ were fused to His and UvATG6 was fused to glutathione *S*-transferase (GST) for the pulldown assays. The results showed that UvBI-1^C-39aa^ and UvBI-1b^C-47aa^ are bound to GST-UvATG6, indicating that the C termini of UvBI-1 and UvBI-1b interacted directly with UvATG6 (Fig. 5B). We also conducted a luciferase (LUC) complementation assay (LCA). UvBI-1 or UvBI-1b was fused to the C-terminal domain of luciferase (cLUC), and UvATG6 was fused to the N-terminal domain of luciferase (nLUC). The chemical luminescence signals were detectable when UvATG6 was coexpressed with UvBI-1 or UvBI-1b in Nicotiana benthamiana (Fig. 5C), indicating the interactions of these proteins. We also found better growth and stronger signals when UvATG6 was coexpressed with UvBI-1 in yeast or N. benthamiana. All of these results demonstrate that UvATG6 interacts with UvBI-1 and UvBI-1b.

### *UvBI-1* and *UvBI-1b* antagonistically regulate mycelial growth and conidiation.

To investigate the function of *UvBI-1b*, we generated gene deletion mutants of *UvBI-1b* and *UvBI-1* as well as *UvBI-1b* double-gene-knockout mutants using protoplast transformation as described below. All deletion mutants were confirmed by PCR using different gene-specific primer pairs (Fig. S8).

**FIG 6:**
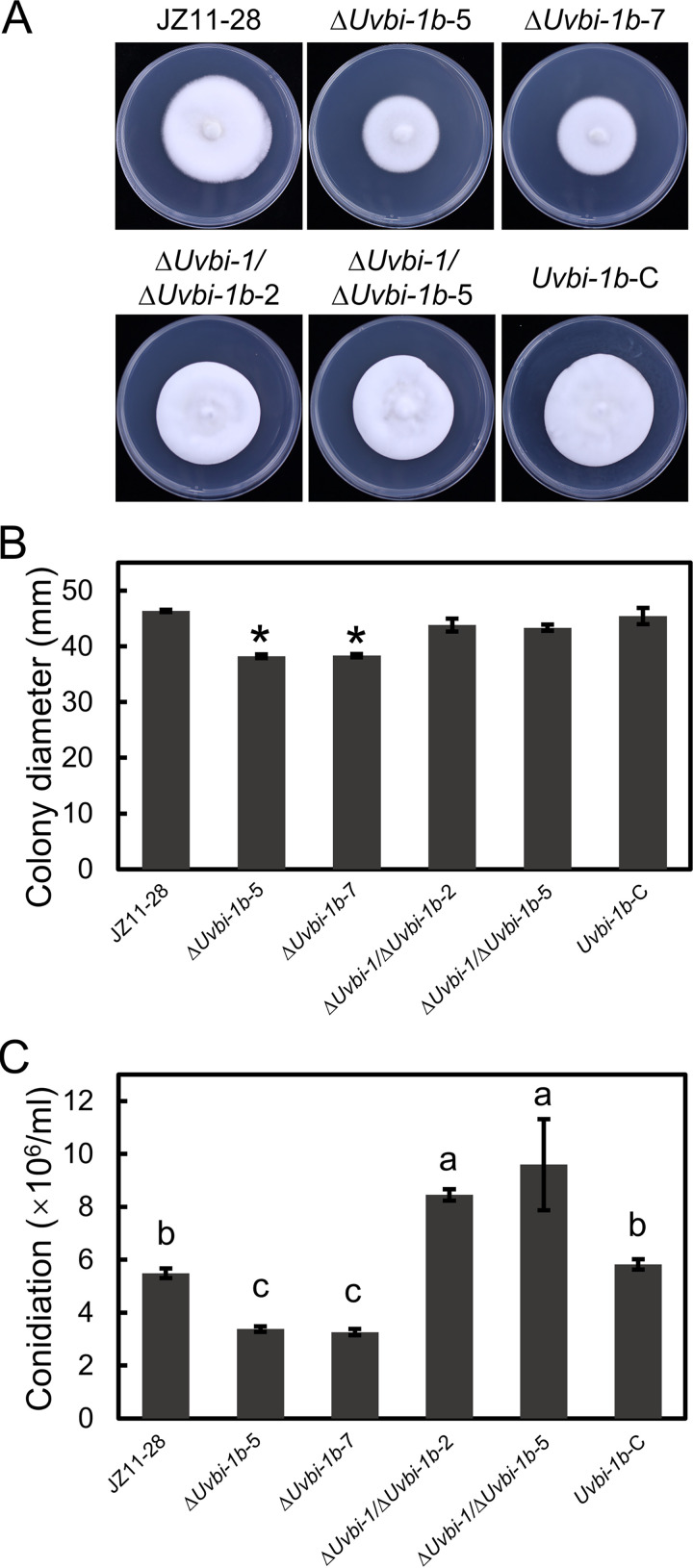
Mycelial development and conidiation of Δ*Uvbi-1b* and Δ*Uvbi-1*/Δ*Uvbi-1b* transformants. (A) Colony morphology of Δ*Uvbi-1b* and Δ*Uvbi-1*/Δ*Uvbi-1b* transformants on PSA, PDA, and YTD. Wild-type strain JZ11-28 and transformant strains were cultured on plates for 14 days at 27°C. (B) Mycelial growth analysis of Δ*Uvbi-1b* and Δ*Uvbi-1*/Δ*Uvbi-1b* transformants. Error bars indicate standard deviations derived from three replicates. (C) Statistical analysis of conidiation. Experiments were performed in three technical replicates with three independent biological replicates. Error bars represent standard deviations, and asterisks represent significant differences (*P < *0.05).

The morphological and growth effects of the deletions of *UvBI-1* and *UvBI-1b* were analyzed on PDA. The colony morphologies of the *UvBI-1b* and double-knockout mutants did not exhibit observable changes. However, the colony diameters of the *UvBI-1b* mutants were decreased, while those of the double mutants were no different (Fig. 6A and B). In addition, the conidiation of the *UvBI-1b* mutants was significantly decreased. In contrast, the conidiation of the double-knockout mutants was increased (Fig. 6C). Due to *UvBI-1* mutants exhibiting higher growth rates and higher levels of production of conidia (27), *UvBI-1* and *UvBI-1b* may antagonistically regulate mycelial growth and conidiation in *U. virens*.

### *UvBI-1* and *UvBI-1b* are involved in stress tolerance.

The tolerance of the *UvBI-1b* mutants and the *UvBI-1* and *UvBI-1b* double mutants to different stressors was determined. The *UvBI-1b* mutants were sensitive to NaCl, SDS, CR, and CFW and insensitive to sorbitol and H_2_O_2_. In addition, the double mutants were more sensitive to the tested stressors than the wild-type strain (Fig. S9). These results suggest that *UvBI-1* and *UvBI-1b* are involved in stress tolerance.

### *UvBI-1b* contributes to the virulence of *U. virens*.

**FIG 7:**
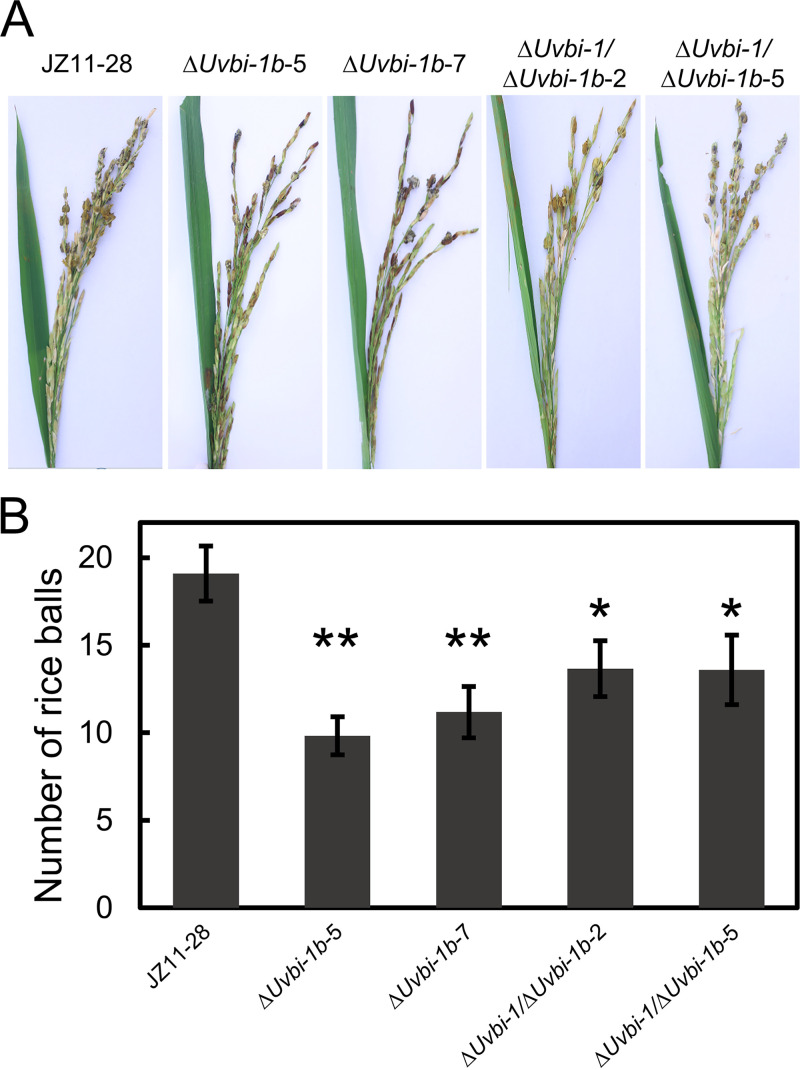
Virulence assay of Δ*Uvbi-1b* and Δ*Uvbi-1*/Δ*Uvbi-1b* transformants. (A) Disease symptom observation of Δ*Uvbi-1b* and Δ*Uvbi-1*/Δ*Uvbi-1b* transformants. A mixed suspension of conidia and hyphae was injected into a single rice panicle at the late booting stage, and the smut balls were observed and photographed 21 days after inoculation. (B) Statistical analysis of the number of smut balls. The number of smut balls in individual rice panicles was measured. At least three independent biological experiments were performed with at least 10 replicates in each test. Error bars represent standard deviations, and asterisks represent significant differences (*, *P < *0.05; **, *P < *0.01).

Previously, we found that *UvBI-1* contributed to the virulence of *U. virens*. To explore whether *UvBI-1b* is involved in virulence, susceptible rice plants were inoculated with the *UvBI-1b* and double mutants. Both mutants exhibited fewer smut balls (Fig. 7), indicating reduced virulence. These results suggest that *UvBI-1b* contributes to virulence.

### UvATG6 and UvBI-1 suppress cell death induced by Bax.

**FIG 8:**
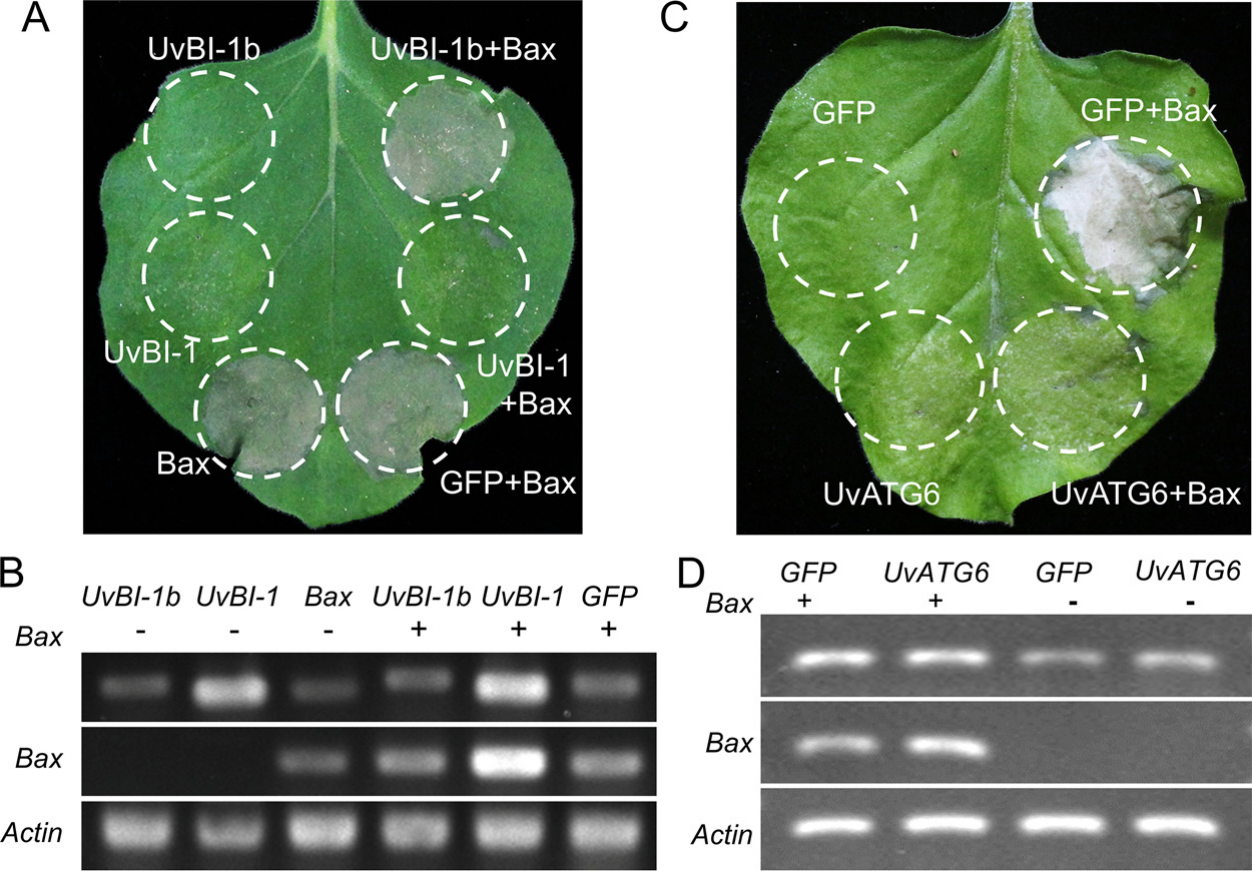
Cell death suppression activity. (A) UvBI-1 can suppress cell death, but UvBI-1b cannot. GFP, UvBI-1, and UvBI-1b were expressed individually or with Bax in N. benthamiana leaves. Cell death was observed and photographed after 7 days. (B) Detection of gene expression by RT-PCR. RNA was extracted 48 h after infiltration, and *UvBI-1b*, *UvBI-1*, *GFP*, and *Bax* were detected. The actin gene of N. benthamiana was amplified as a control. (C) UvATG6 suppresses cell death induced by Bax. GFP and UvATG6 were expressed individually or with Bax in N. benthamiana leaves. Cell death was observed and photographed after 7 days. (D) Detection of *UvATG6* expression by RT-PCR. RNA was extracted 48 h after infiltration, and *UvATG6*, *GFP*, and *Bax* were detected. The actin gene of N. benthamiana was amplified as a control. The experiments were performed in three independent biological replicates with three technical replicates each time.

UvBI-1b contains a Bax inhibitor 1 domain, and UvBI-1 can suppress cell death induced by Bax (27). UvBI-1b was coexpressed with Bax in N. benthamiana to explore whether UvBI-1b has the same ability to suppress cell death. The results showed that UvBI-1b could not suppress cell death induced by Bax, which is identical to the results with the GFP negative control (Fig. 8A). RT-PCR was used to determine the expression levels of all of the genes (Fig. 8B). These results suggest that UvBI-1 and UvBI-1b exhibit distinctive activities of cell death suppression. ATG6 is critical for autophagy and interacts with UvBI-1 and UvBI-1b. To investigate whether UvATG6 suppresses cell death induced by Bax, we coexpressed UvATG6 and Bax in N. benthamiana leaves (Fig. 8C). The results showed that the UvATG6 could suppress Bax-induced cell death and was involved in apoptosis. All of the genes were detectable using RT-PCR (Fig. 8D). All of these results indicate that UvATG6 and UvBI-1 exhibit the same ability for cell death suppression as that induced by Bax.

### UvBI-1 negatively regulates the expression of ATGs in *U. virens*.

**FIG 9:**
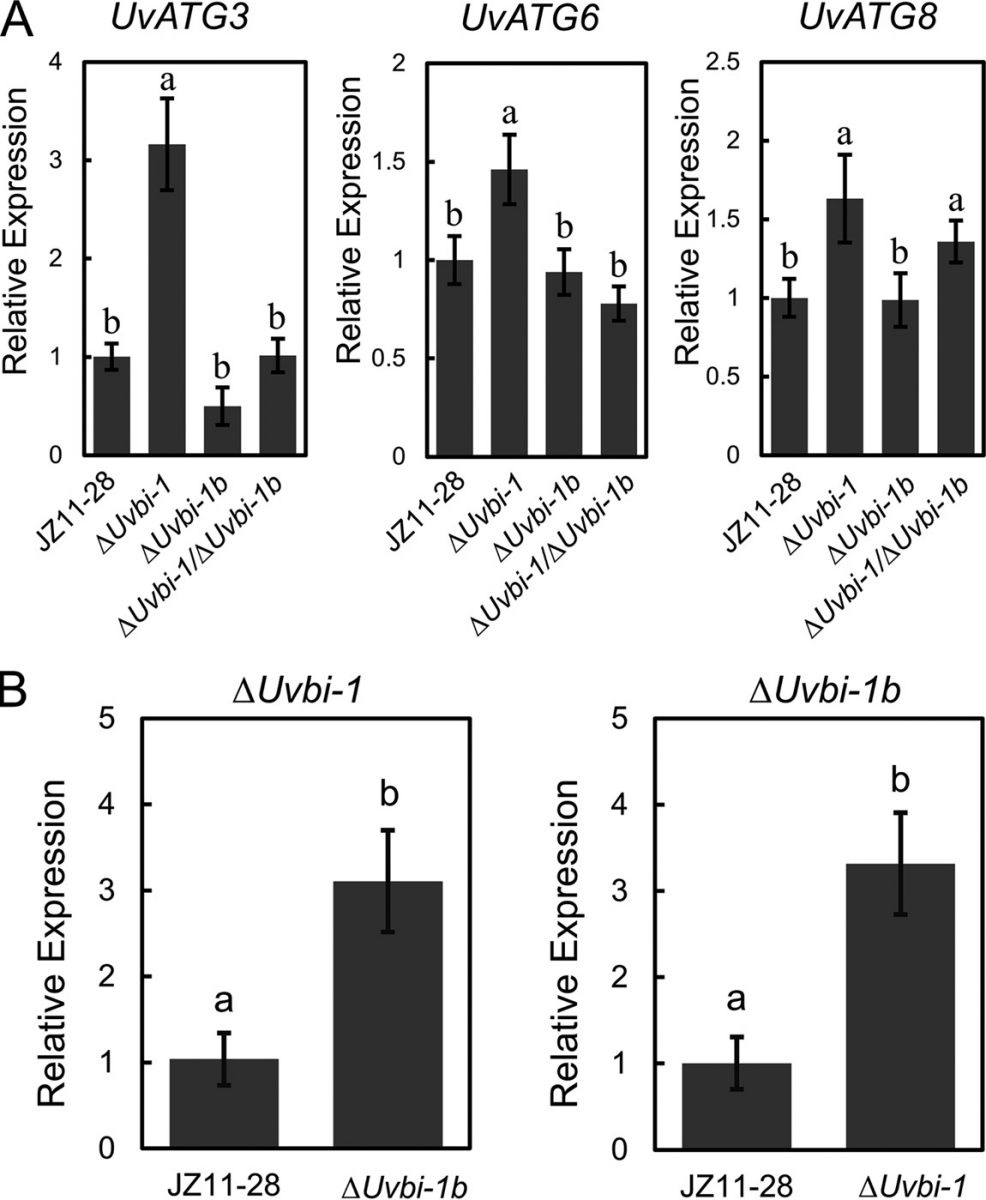
Expression of autophagy-related and Bax inhibitor 1 genes in Δ*Uvbi-1b* and Δ*Uvbi-1*/Δ*Uvbi-1b* transformants. (A) Expression levels of autophagy-related genes in Δ*Uvbi-1*, Δ*Uvbi-1b*, and Δ*Uvbi-1*/Δ*Uvbi-1b* transformants. The mycelial RNAs from the strains were extracted, and gene expression levels were determined using RT-qPCR. The expression level in wild-type strain JZ11-28 was normalized to a value of 1. The letters represent statistical significance (*P < *0.05). (B) Expression levels of *UvBI-1* and *UvBI-1b* in the Δ*Uvbi-1b* and Δ*Uvbi-1*strains. The expression level in wild-type strain JZ11-28 was normalized to a value of 1. The experiments were performed in three independent biological replicates with three technical replicates each time. The letters represent statistical significance (*P < *0.05).

To investigate the effect of BI-1 proteins on autophagy in *U. virens*, the expression levels of three ATGs in the *UvBI-1*, *UvBI-1b*, and double mutants were determined. *UvATG3*, *UvATG6*, and *UvATG8* were upregulated in the *UvBI-1* mutants. However, there was no difference in the expression of these genes in the *UvBI-1b* and double mutants except for *UvATG8*, which was upregulated in the double mutants (Fig. 9A). These results suggest that UvBI-1 negatively regulates the expression of ATGs in *U. virens*. We also determined the expression levels of *UvBI-1b* in *UvBI-1* mutants and *UvBI-1* in *UvBI-1b* mutants to determine the mutual influences of *UvBI-1* and *UvBI-1b*. The results showed that *UvBI-1b* and *UvBI-1* were upregulated in the counterpart mutants (Fig. 9B), indicating that they can inhibit each other. We investigated the expression levels of *UvBI-1* and *UvBI-1b* in the *UvATG6* mutants. The expression level of *UvBI-1* was no different, but that of *UvBI-1b* was upregulated (Fig. S10).

## DISCUSSION

Autophagy and apoptosis are critical for development, cellular homeostasis, and physiological and pathological processes. The ATG6 and BI-1 proteins are evolutionarily conserved across eukaryotes and have essential functions in apoptosis and autophagy, respectively. In this study, we found that UvATG6 played essential roles in autophagy, growth, conidiation, and virulence. Furthermore, we discovered that UvATG6 could interact with both Bax inhibitor 1 proteins in *U. virens* and that these proteins may antagonistically regulate growth, conidiation, and stress responses.

ATG6 is involved in autophagy and apoptosis by interacting with multiple proteins (9). In Drosophila melanogaster, ATG6 is required for multiple vesicle trafficking processes and hematopoiesis (28). *Beclin 1*/*ATG6* in N. benthamiana is essential for plant innate immunity and negatively regulates programmed cell death (29). The same phenomenon was observed in Arabidopsis thaliana as plants with *AtATG6* knockdown failed to limit the spread of resistance- and disease-associated cell death (30). Plant Bax inhibitor 1 interacts with ATG6 to regulate autophagy and programmed cell death (21).

The autophagy process in phytopathogenic fungi participates in the interaction between pathogens and their host plants, and many of the ATGs, including ATG6, contribute to virulence and other biological processes (31). For example, *PsAtg6a* in Phytophthora sojae contributes to sporulation and virulence (32). *FgATG6* is required for growth and virulence in Fusarium graminearum (33). In Peronophythora litchii, *PlATG6a* is involved in growth, asexual reproduction, and stress tolerance (34). In addition, several ATGs in *U. virens*, including *UvATG8*, *UvATG7*, and *UvATG14*, have been characterized as being involved in growth, conidiation, and virulence (24–26). In this study, we found that *UvATG6* was required for autophagy in *U. virens* and that the deletion of *UvATG6* leads to defects in growth, conidial production and germination, and virulence, indicating its essential role in these processes.

BI-1-deficient mouse cells exhibit hypersensitivity to apoptosis induced by ER stress agents, which could be prevented by BI-1 overexpression (19). In Procambarus clarkii, the hemocyte programmed cell death rate was significantly increased, and the replication of white spot syndrome virus declined when *PcBI-1* was silenced (35), indicating that BI-1 is involved in biotic stress. Plants that overexpress AtBI-1 can suppress cell death induced by biotic and abiotic stresses, including fungal pathogens, fungal elicitors, hydrogen peroxide, and salicylic acid (36, 37). BI-1 in wheat contributes to resistance, probably by interacting with an aquaporin, TaPIP1 (38, 39). In N. benthamiana, the overexpression of *BI-1* increased autophagic activity. Conversely, the silencing of *BI-1* reduced autophagic activity and enhanced *N* gene-mediated cell death, indicating prosurvival and prodeath roles in the different physiological processes (21). Little is known about the roles of *BI-1* in filamentous fungi. The silencing of *Ss-Bi1* in Sclerotinia sclerotiorum was shown to increase its sensitivity to heat and ER stress and reduce its virulence (40). In the pathogenic insect fungus Metarhizium robertsii, MrBI-1 regulates heat tolerance, apoptosis-like cell death, and virulence (41). We previously found that UvBI-1 could suppress Bax-induced cell death and negatively regulate growth and conidiation. In this study, we found that UvBI-1b cannot suppress cell death, and *UvBI-1* and *UvBI-1b* probably play antagonistic roles in regulating growth and conidiation. The expressions of *UvBI-1* in the *UvBI-1b* mutant and *UvBI-1b* in the *UvBI-1* mutant were upregulated. This finding indicated the antagonistic functions of *UvBI-1* and *UvBI-1b*, probably by affecting the expression of each other’s genes.

In this study, UvATG6 could suppress cell death induced by Bax, indicating its function in inhibiting programmed cell death. Although UvBI-1 and UvBI-1b exhibit different cell death-inhibitory activities, they can interact with UvATG6, suggesting cross talk between autophagy and apoptosis in *U. virens*. We also found that the expression of *UvBI-1b* was upregulated in the *UvATG6* mutant. In addition, *UvATG3*, *UvATG6*, and *UvATG8* were upregulated in the *UvBI-1* mutant. ATG8 is a ubiquitin-like protein required for the formation of double-membrane autophagosomes and therefore plays a critical role in the autophagy process (42, 43). In Δ*Uvbi-1*/Δ*Uvbi-1b* transformants, the expression levels of *UvATG3* and *UvATG6* were no different, but *UvATG8* was upregulated. These results indicate that *UvBI-1* is a negative regulator of autophagy and probably plays more important roles than *UvBI-1b*.

In summary, UvATG6 is critical for autophagy and contributes to growth, conidiation, and virulence. UvBI-1 and UvBI-1b seem to antagonistically regulate the growth and conidiation of *U. virens*, and both contribute to virulence. Furthermore, UvATG6 interacts with UvBI-1 and UvBI-1b, indicating cross talk between autophagy and apoptosis. Our results suggest that Bax inhibitor 1 proteins and UvATG6 are essential for the growth, conidiation, and virulence of *U. virens* and give clues for studying other phytopathogenic fungi.

## MATERIALS AND METHODS

### Sequence analysis.

The gene and protein data used in this study were downloaded from the National Center for Biotechnology Information (NCBI) (https://www.nih.gov/). Protein structure predictions were performed using SMART (http://smart.embl-heidelberg.de/). Sequence alignments were performed using BioEdit, and phylogenetic analyses were performed using MEGA 7.0 based on maximum likelihood.

### Fungal strains and culture media.

*U. virens* strains JS60-2 and JZ11-28 were used as wild-type strains. JS60-2 was used for UvATG6 knockout, and JZ11-28 was used for UvBI-1 and UvBI-1b knockouts (see Table S1 in the supplemental material). The colony diameter of JS60-2 after 7 days of culture on PSA (200 g/L potato, 20 g/L sucrose, and 15 g/L agar) was 35 mm, and that of JZ11-28 was 46 mm. The level of conidiation of JS60-2 was 20 × 10^6^ conidia/mL, and that of JZ11-28 was 5.5 × 10^6^ conidia/mL. The *U. virens* strains were cultured on PSA, potato dextrose agar (PDA) (200 g/L potato, 20 g/L d-glucose, and 15 g/L agar), and YTD medium [1 g/L yeast extract, 1 g/L tryptone, 10 g/L d-(+)-glucose, and 15 g/L agar] at 27°C for the assessment of colony growth.

For the conidial concentrations, the strains were incubated in potato sucrose broth (PSB) (PSA without agar) medium at 160 rpm at 27°C for 7 days. Next, the conidial concentrations were measured using a hemocytometer. For the germination test, the conidia were spread onto PSA and WA (water and agar only) plates, three regions were selected on one plate, and 100 conidia were observed within each region. These were used to count the number of germinating conidia.

To test stress tolerance, we evaluated *U. virens* strains after 14 days of growth on PSA medium containing 0.4 M NaCl, 0.7 M sorbitol, 0.04% sodium dodecyl sulfate (SDS), 200 μg/mL CR, 120 μg/mL CFW, or 0.03% H_2_O_2_. The inhibition rate was calculated as follows: inhibition rate (%) = [(average colony diameter on PSA − average colony diameter on PSA with a stress agent)/average colony diameter on PSA] × 100. All of the experiments were repeated three times, with three replicates each time.

### Gene expression analysis.

Total RNA samples were isolated using TRIzol reagent (Invitrogen), and the cDNA was synthesized using a HifairII first-stand cDNA synthesis kit (Yeasen, Shanghai, China). The reaction mixtures were diluted 10 times and used as the templates. Quantitative real-time PCR (qPCR) was performed using a CFX96 real-time PCR detection system (Bio-Rad Laboratories Inc.). The relative expression level of the gene was calculated using the 2^−ΔΔ^*^CT^* method, with the β-tubulin gene as the reference gene. Data from three biological replicates were used to calculate the means and standard deviations. RT-PCR was conducted with 30 cycles. All primers used in this assay are listed in Table S1.

### Transient expression in N. benthamiana.

The coding sequences (CDSs) were amplified and cloned into the vector pGR107 using a one-step cloning kit (Yeasen, Shanghai, China). The constructs were then introduced into Agrobacterium tumefaciens strain GV3101 by electroporation. With the strain containing GFP as the negative control, those containing recombinant vectors were cultured in Luria-Bertani (LB) medium (10 g/L tryptone, 10 g/L yeast extract, 5 g/L NaCl) at 30°C (with shaking) at 200 rpm for 24 h. The cultures were harvested by centrifugation, washed with 10 mM MgCl_2_ three times, and resuspended in 10 mM MgCl_2_ to achieve a final optical density at 600 nm (OD_600_) of 0.3. Next, the A. tumefaciens cell suspension was infiltrated into N. benthamiana leaves using a syringe without a needle. After 24 h, the strain containing Bax was infiltrated.

### Targeted gene deletion and complementation.

For CRISPR-Cas system vector construction, a short cassette was generated by annealing the sense and antisense oligonucleotides (Table S2). This cassette was inserted into the pCas9:tRp-gRNA vector digested with Esp3I. The combination of CRISPR-Cas9 and the homologous recombination method generated gene deletion mutants. Briefly, fragments of 1.2-kb upstream and 1.3-kb downstream flanking sequences of *UvATG6* or those of 0.8-kb upstream and 0.8-kb downstream flanking sequences of *UvBI-1b* were amplified from the genomic DNA of *U. virens* using the primers from the first round of amplification. Next, the 5′ and 3′ parts of the hygromycin B phosphotransferase gene (*hph*) were amplified using the primer pair HYG-F and H3 and the primer pair H2 and HYG-R, respectively. The upstream and downstream flanking sequences were joined with the 5′ and 3′ parts of the *hph* gene, respectively. The CRISPR vector and recombinant fragments were introduced into protoplasts using protoplast-mediated transformation. These fragments of *UvATG6* were introduced into wild-type strain JS60-2 to generate *UvATG6* mutants, and the JZ11-28 strain was used to generate *UvBI-1b* mutants. The homologous recombination fragments and CRISPR vectors of the *UvBI-1* and *UvBI-1b* genes were transformed simultaneously into wild-type strain JZ11-28 to obtain the *UvBI-1* and *UvBI-1b* double-gene-knockout mutants.

To generate complemented strains, the full-length genomic sequence with 1.5-kb upstream and 0.5-kb downstream flanking sequences of the gene was amplified and inserted into either the pNeo vector (*UvATG6*) or the pCETNS4 vector (*UvBI-1b*) that contains Geneticin resistance genes. Next, the vectors were transformed into the mutants using protoplast-mediated transformation.

### Yeast two-hybrid assay.

Plasmid pGBKT7, containing UvATG6, and plasmid pGADT7, with either UvBI-1^C-39aa^ or UvBI-1b^C-47aa^, were cotransformed into yeast strain AH109. The strain that coexpressed pGADT7-T and pGBKT7-53 was used as a positive control, and those that coexpressed empty pGADT7 plasmids were used as negative controls. After PCR confirmation, the colonies were cultured (with shaking) at 200 rpm at 30°C for 48 h. When the OD_600_ reached 0.4 to 0.5, the cultured yeast cells were centrifuged and resuspended in double-distilled water (ddH_2_O). Next, the yeast cell suspension was diluted and incubated on synthetic defined (SD)/−Trp−Leu−His−Ade medium with and without X-α-Gal (50 μL [4 μg/mL], spread on top of the medium) or 10 μM DTT at 30°C for 3 days.

### Pulldown assay.

The pGEX-4T-2 vector (GST) fused with UvATG6 and pET-32a vectors (His) fused with UvBI-1^C-39aa^ or UvBI-1b^C-47aa^ were transformed into Escherichia coli BL21(DE3). A single colony was incubated in LB medium to an OD_600_ value of 0.4 to 0.6. Next, 0.1 mM isopropyl-β-d-thiogalactopyranoside (IPTG) was added for protein expression induction at 30°C for 4 h. The bacteria were collected and resuspended in phosphate-buffered saline (PBS) (8 g/L NaCl, 0.2 g/L KCl, 1.42 g/L Na_2_HPO_4_, and 0.27 g/L KH_2_PO_4_ [pH 7.4]). The supernatant was crushed using an ultrasonic processer to obtain the protein solution.

His–UvBI-1^C-39aa^, His–UvBI-1b^C-47aa^, and His (pET-32a-expressed protein) were incubated with His beads at 4°C for 8 h and then incubated with the GST-UvATG6 protein for another 8 h. The beads were washed with 1× PBS containing 1% Triton X-100 five times, and proteins were separated by SDS-PAGE (TGX stain-free fast cast acrylamide kit, 12%; Bio-Rad, USA). Immunoblot assays were performed with an anti-His (Proteintech, Wuhan, China) or an anti-GST (Proteintech, Wuhan, China) antibody.

### Luciferase complementation imaging (LCI) assay.

UvATG6 was fused with the N terminus of LUC (ATG6:nLUC), and UvBI-1 or UvBI-1b was fused with the C terminus of LUC (cLUC:UvBI-1 or cLUC:UvBI-1b, respectively). The plasmids were transformed into A. tumefaciens GV3101. The *Agrobacterium* cells were infiltrated into N. benthamiana leaves. After 48 h, 1 mM d-luciferin (Yeasen, Shanghai, China) was sprayed onto the leaves before the relative LUC activity test. Luminescence was captured using a Berthold (Germany) Night Shade LB 985 instrument after a 5-min exposure.

### Generation of the GFP-UvATG8 strain and autophagy analysis.

To generate GFP-UvATG8-expressing strains, 1 kb of the promoter region of *UvATG8*, the enhanced GFP (eGFP) gene, and the CDS of *UvATG8* were inserted into pKNRG823 (Table S2). The plasmid was introduced into wild-type strain JS60-2 or the Uvatg6 knockout mutant by protoplast-mediated transformation. The transformants were confirmed by PCR and epifluorescence microscopic observations.

For the induction of autophagy, the GFP-UvATG8 strains were cultured in PSB medium for 3 days, followed by starvation in SD−N medium (yeast nitrogen base without ammonium sulfate and with 1.7 g/L amino acids and 20 g/L glucose) for 6 h. The mycelia cultured in PSB medium were used as the controls. The mycelia were collected by centrifugation at 10,000 rpm for 1 min, washed three times with sterile water, and suspended in 1 mL 0.2 M PBS (51.58 g/L Na_2_HPO_4_ and 8.74 g/L NaH_2_PO_4_ [pH 7.4]). Two microliters of 50 mM monodansylcadaverine (MDC) (17 mg of MDC dissolved in 1 mL of methanol) was added to the suspension, which was mixed well and immediately kept in the dark at room temperature for 45 min. The treated mycelia were collected by centrifugation at 10,000 rpm for 1 min and washed three times with 0.2 M PBS. The samples were observed by confocal microscopy (SP8; Leica), and MDC and eGFP excitations were performed at 405 nm (emission wavelengths of 415 to 450 nm) and 488 nm (emission wavelengths of 498 to 540 nm), respectively. The mycelial protein was extracted using 1× PBS containing 1 mM phenylmethylsulfonyl fluoride (PMSF) and separated on an SDS-polyacrylamide gel electrophoresis (PAGE) gel. An anti-GFP antibody (Proteintech, Wuhan, China) and horseradish peroxidase (HRP)-conjugated goat anti-mouse IgG (Proteintech, Wuhan, China) were used for the blotting processes. The samples were also hybridized with an anti-glyceraldehyde-3-phosphate dehydrogenase (GAPDH) monoclonal antibody (Dia-An, Wuhan, China) for an internal reference. ImageJ was used to analyze the levels of gray bands.

### Plant inoculation assays.

A susceptible rice cultivar (Wanxian-98) was used for inoculation. The plugs were placed into PSB and shaken at 160 rpm at 27°C for 7 days, and the cultured fungi were crushed. The conidia were measured and adjusted to 5 × 10^6^ conidia/mL with PSB. Approximately 5 mL of the conidial and hyphal suspension in PSB was injected into a single rice panicle from the middle to the upper portions at the late booting stage (3 to 5 days before heading) using a syringe. Inoculated plants were maintained in a greenhouse at 27°C with 90 to 100% relative humidity (RH) for 7 days and then placed at 27°C with 80% RH until false smut balls appeared. The symptoms were observed, and the number of smut balls was measured 21 days after inoculation. All of the experiments described above were performed at least three times independently, with at least 10 replicates in each test.
