# Supplementary material for: UvATG6 Interacts with BAX Inhibitor 1 Proteins and Plays Critical Roles in Growth, Conidiation, and Virulence in Ustilaginoidea virens
Source: Microbiol Spectr. 2023 Apr 27;11(3):e04898-22. doi: 10.1128/spectrum.04898-22 (PMC10269921; doi:10.1128/spectrum.04898-22)
Supplement: Supplemental file 1 — Supplemental material. Download spectrum.04898-22-s0001.pdf, PDF file, 1.8 MB [file spectrum.04898-22-s0001.pdf]

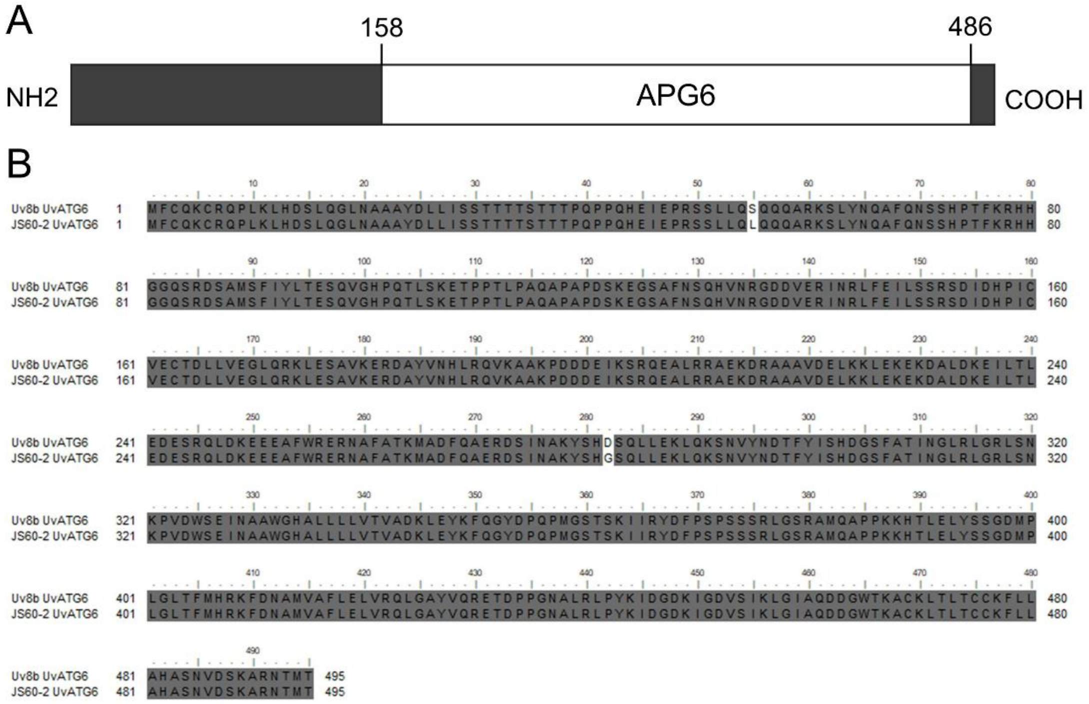

Fig. S1 Protein structure analysis and sequence alignment of UvATG6

(A) Protein structure analysis of UvATG6. (B) Protein sequence alignment of UvATG6 from Uv8b and JS60-2 strains. Specific primers were used to amplify UvATG6 from the cDNA of strain JS60-2.

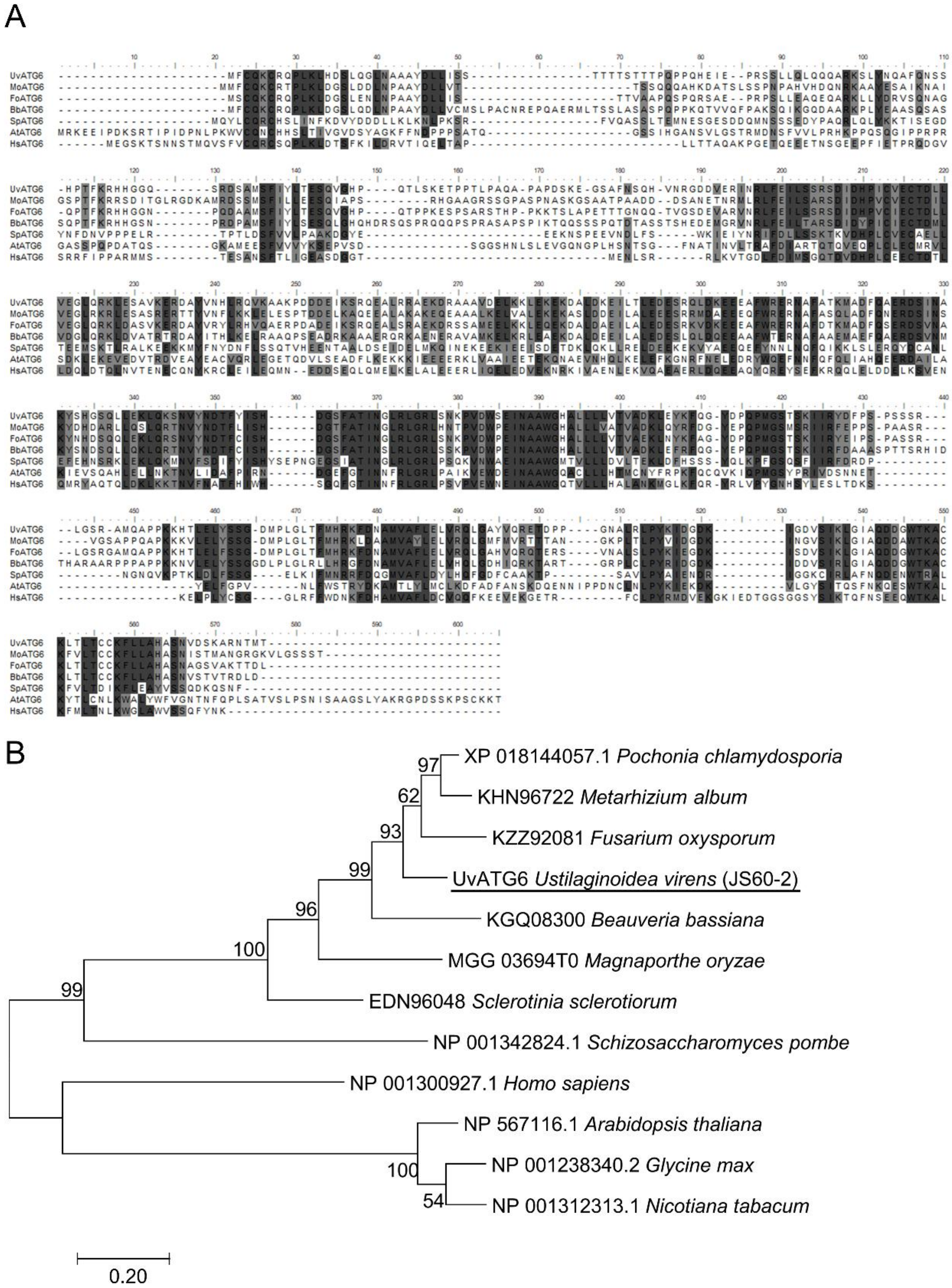

Fig. S2 Sequence alignment and phylogenetic analysis of UvATG6.

(A) Sequence alignment of UvATG6 with other orthologs. The ATG6 from *U. virens* JS60-2 strain (UvATG6), *Magnaporthe oryzae* (MoATG6, MGG03694), *Fusarium oxysporum* (FoATG6, KZZ92081), *Beauveria bassiana* (BbATG6, KGQ08300), *Schizosaccharomyces pombe* (SpATG6, NP001342824.1), *Arabidopsis thaliana* (AtATG6, NP567116.10), and *Homo sapiens* (HsATG6, NP001300927.1) were collected and aligned. (B) Phylogenetic analysis of UvATG6 with different orthologs.

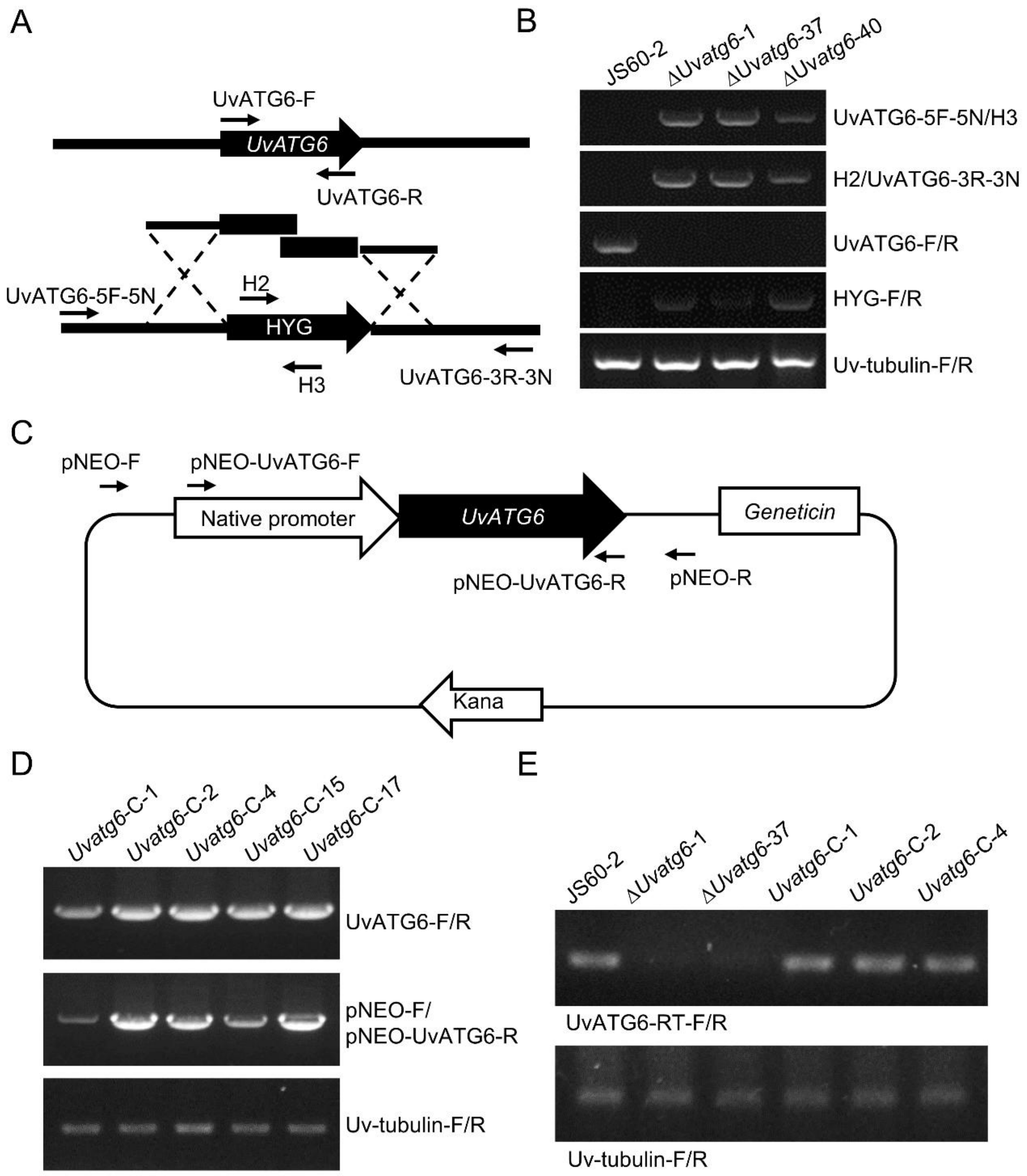

Fig. S3 *UvATG6* knockout and complemented transformants validation.
(A) Schematic diagram of *UvATG6* gene knockout strategy. *UvATG6* was replaced with hygromycin B phosphotransferase gene (*hph*) by homologous recombination. (B) Validation of *UvATG6* knockout mutants. Specific primers shown in (A) were used to verify the knockout of *UvATG6* by PCR. (C) *UvATG6* complementary strategy. (D) Validation of UvATG6 complemented transformants. Specific primers showed in (C) were used to verify the complement of *UvATG6* by PCR. (E) Expression of *UvATG6* in knockout and complemented strains. The RNA from mycelia was extracted, and RT-PCR was used to detect gene expression.

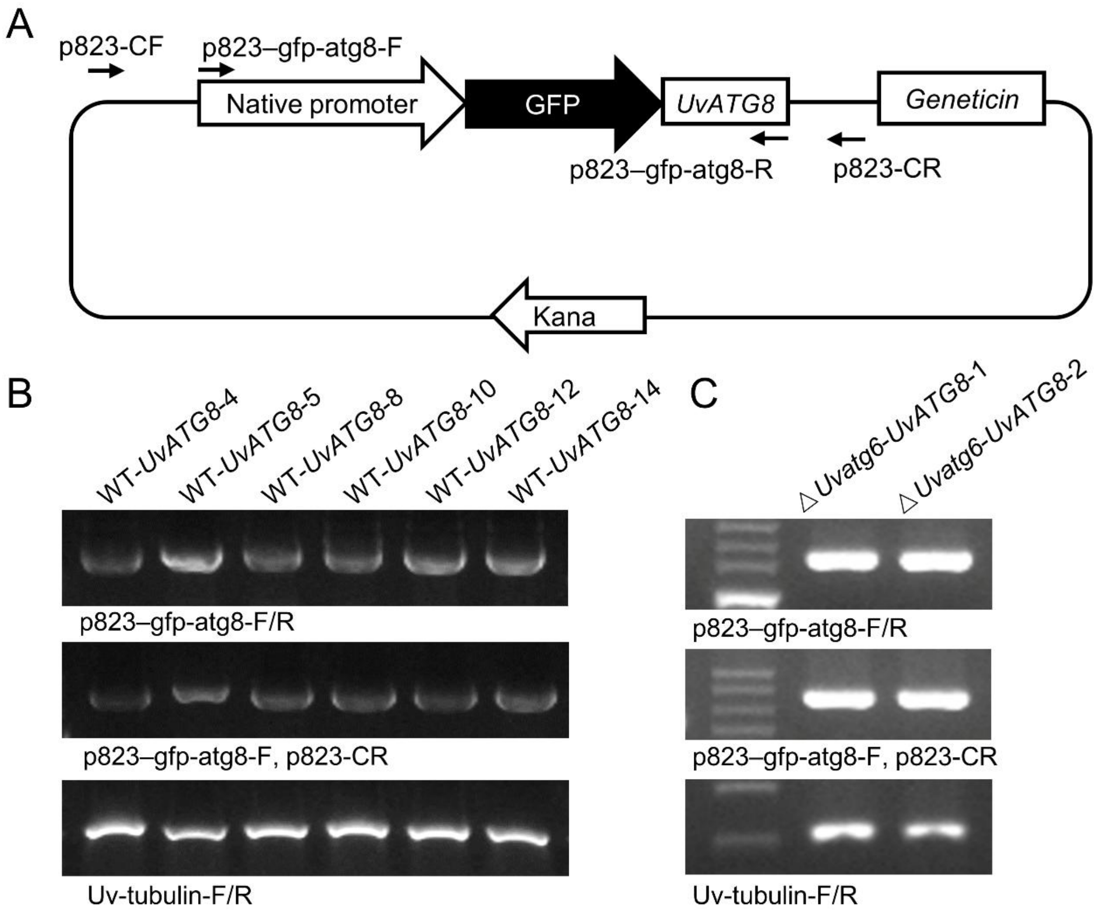

Fig. S4 The validation of GFP-UvATG8 expressed transformants.

(A) Primer positioning used in the validation experiments. (B) Validation of GFP-UvATG8 expressed transformants in wild-type JS60-2 background. WT indicates wild-type JS60-2 strain. (C) Validation of GFP-UvATG8 expressed transformants in *Uvatg6* mutant background.

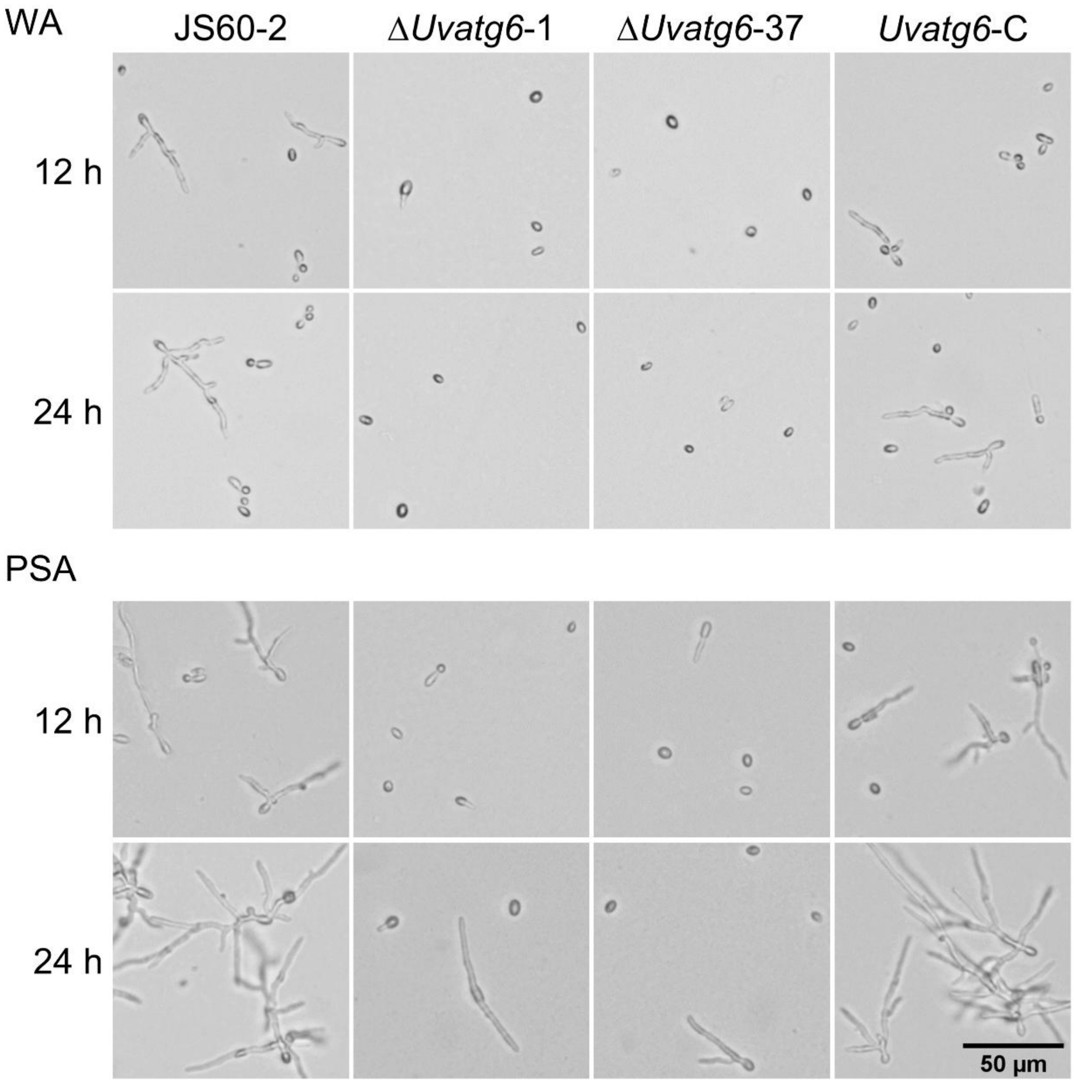

Fig. S5 Conidial germination of Δ*UvATG6* transformants.

The conidia were spread on water agar (WA) and PSA plates, and the germination morphology was observed at 12 and 24 h.

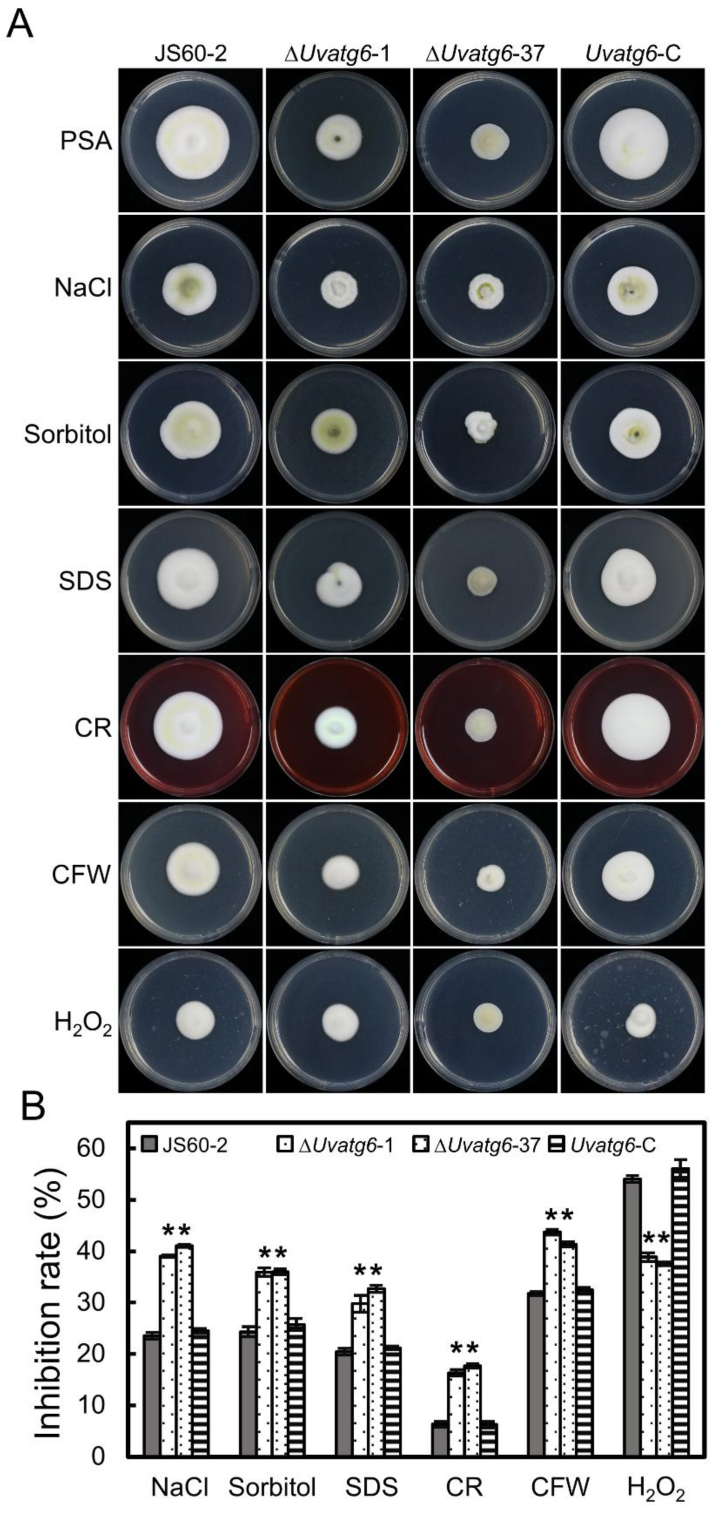

Fig. S6 Sensitivity of Δ*Uvatg6* transformants to different stressors.

(A) Colonial morphology of Δ*Uvatg6* transformants under different stressors. The strains were cultured on PSA containing 0.4 M NaCl, 0.7 M sorbitol, 0.04% SDS, 200 μ g/mL CR, 120 μg/mLCFW, and 0.03% H_2O_2. (B) Growth inhibition rate analysis under different stressors. The experiments were performed in three independent biological replicates with three technical replicates each time. Error bars represent standard deviation, and asterisks represent significant differences ($P < 0.05$).

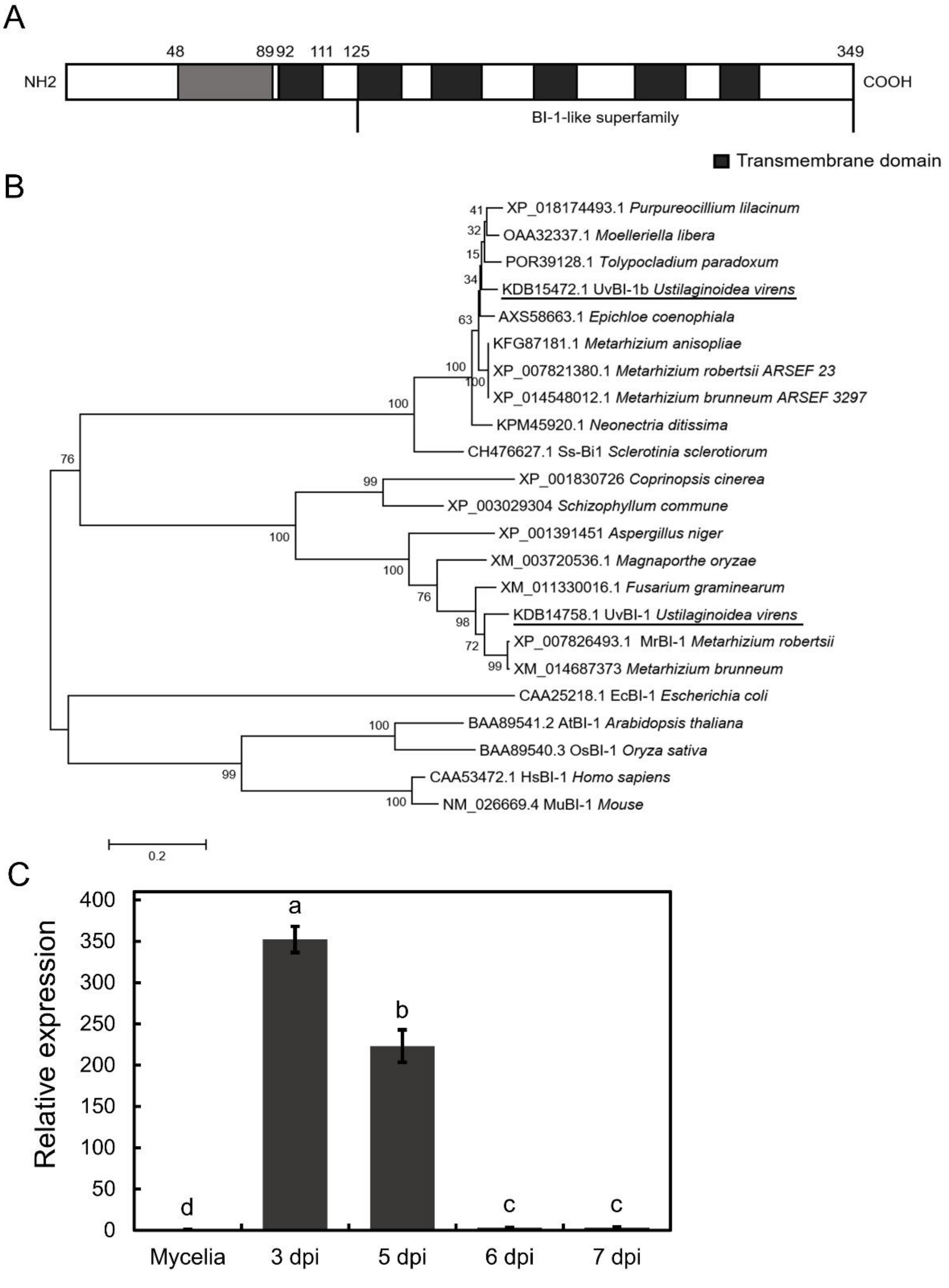

Fig. S7 Sequence analysis and expression pattern of *UvBI-1b*.
(A) The protein domain analysis of UvBI-1b. (B) Phylogenetic analysis of UvBI-1b with different orthologs. (C) The expression level of *UvBI-1b* at infection stages. The susceptible rice cultivar (Wanxian-98) was inoculated by the *U. virens* strain JZ11-28. The expression of UvBI-1b was detected using qRT-PCR for the samples, including JZ11-28 mycelia, at 3, 5, 6, and 7 days post-inoculation (dpi). The fragment of the *β-tubulin* gene from *U. virens* was used as an internal reference, and the expression level of mycelia was normalized to one. Error bars represent standard deviations derived from three replicates. The letters represent statistical significance ($P < 0.05$).

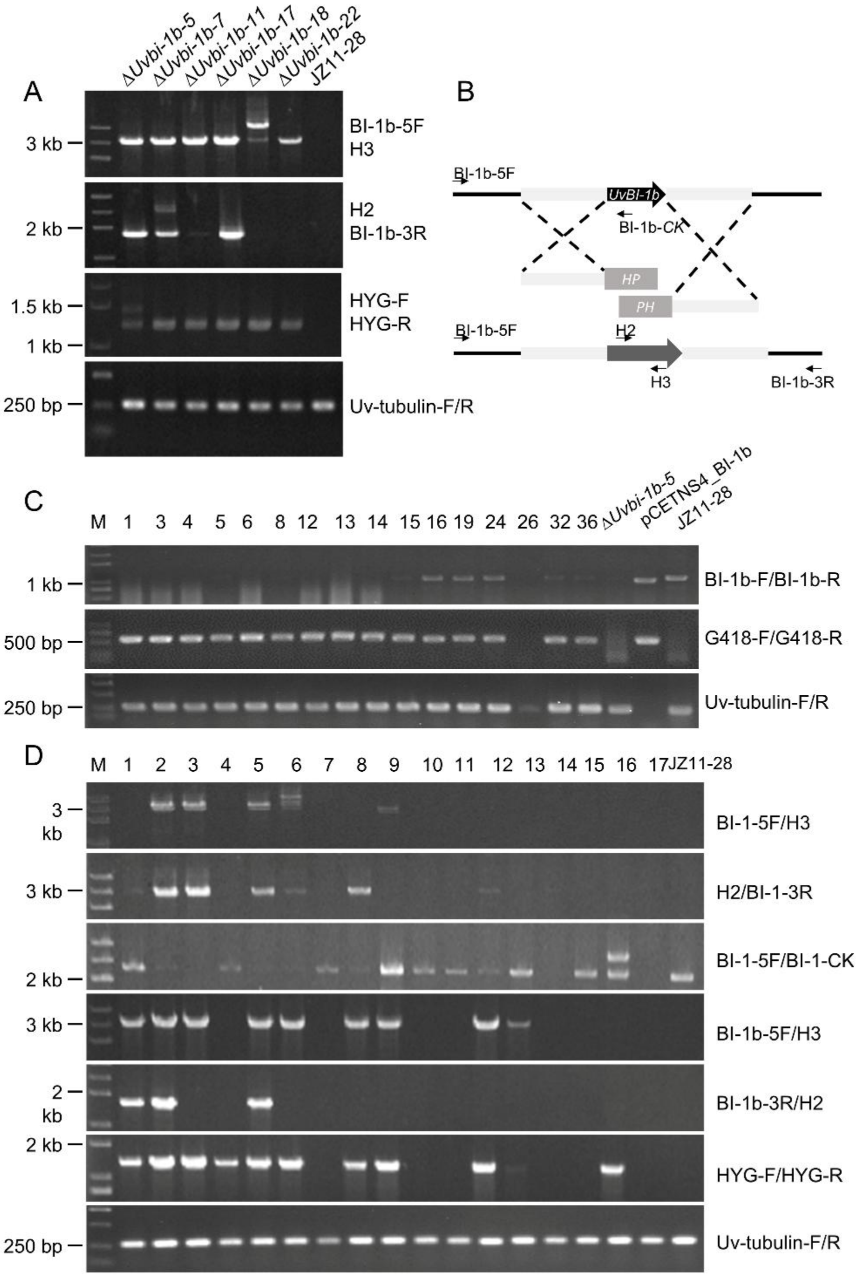

Fig. S8 Gene knockout strategy and transformants validation.
(A) Verification of *UvBI-1b* knockout mutants. Positive transformants were verified by PCR using different primer pairs, as shown in B. M: marker, HYG-F, and HYG-R are primers for hygromycin B, amplifying *β-tubulin* fragments as a control. (B) Schematic diagram of *UvBI-1b* gene knockout strategy. The gray rectangles represent fragments of homologous recombination. (C) Verification of *UvBI-1b* complemented mutants. The numbers indicate different *UvBI-1b* complemented transformants. G418-F and G418-R are primers for the amplification of the geneticin resistance gene. The *β-tubulin* fragment is amplified to identify DNA quality. (D) PCR validation of double knockout transformants. The numbers indicate different transformants. The *UvBI-1* and *UvBI-1b* genes were simultaneously knocked out based on homologous recombination, and PCR verified the replacements of the two genes with the indicated primers.

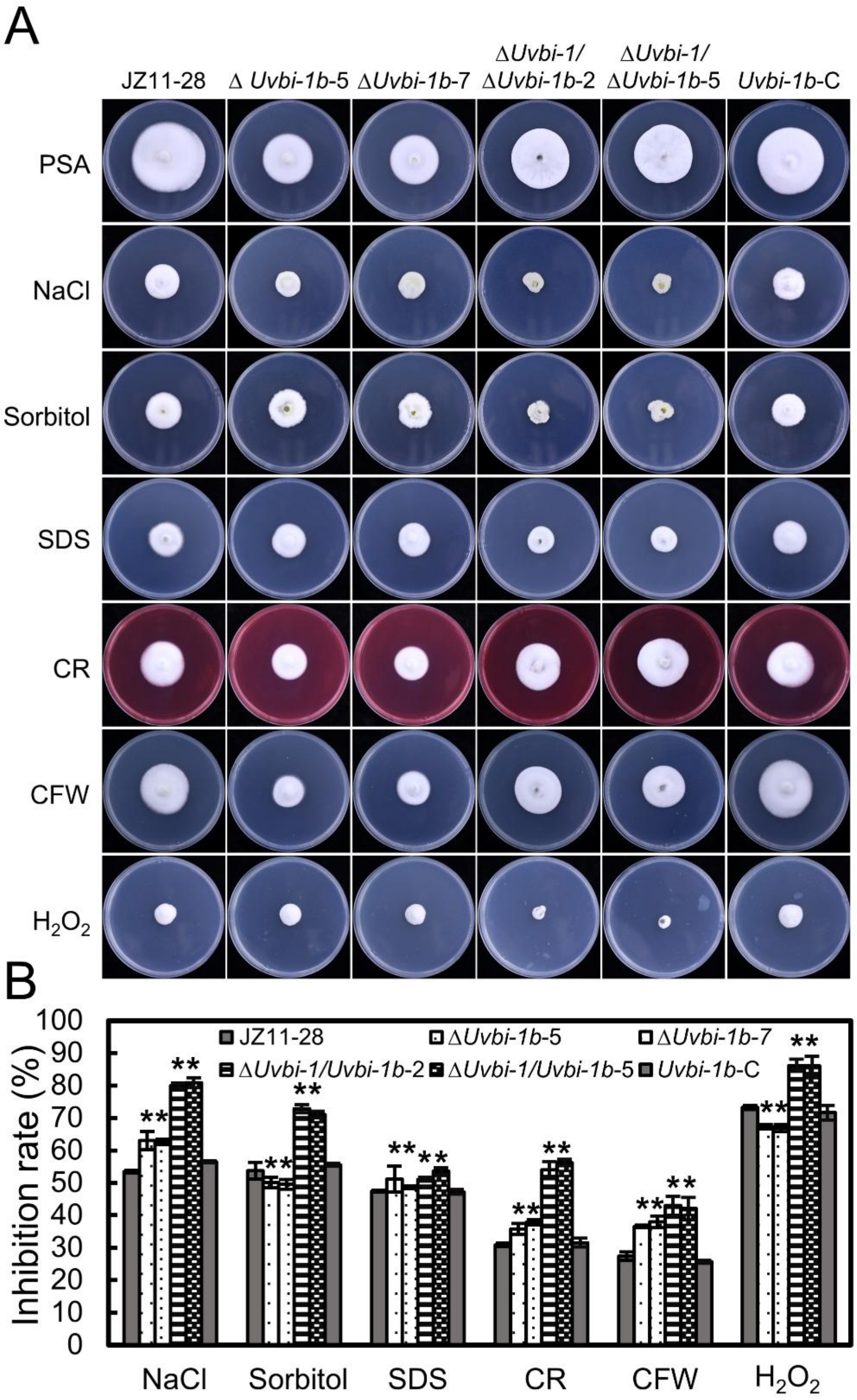

Fig. S9 Sensitivity of Δ*Uvbi-1b*, Δ*Uvbi-1*/Δ*Uvbi-1b* transformants to different stressors. (A) Colonial morphology of Δ*Uvbi-1b*, Δ*Uvbi-1*/Δ*Uvbi-1b* transformants under different stressors. The strains were cultured on PSA containing 0.4 M NaCl, 0.7 M Sorbitol, 0.03% SDS, 120 μg/mL CR, 120 μg/mL CFW, and 0.03% H_2O_2. (B) Colony growth inhibition rate analysis under different stressors. The experiments were performed in three independent biological replicates with three technical replicates each time. Error bars represent standard deviation, and asterisks represent significant differences ($P < 0.05$).

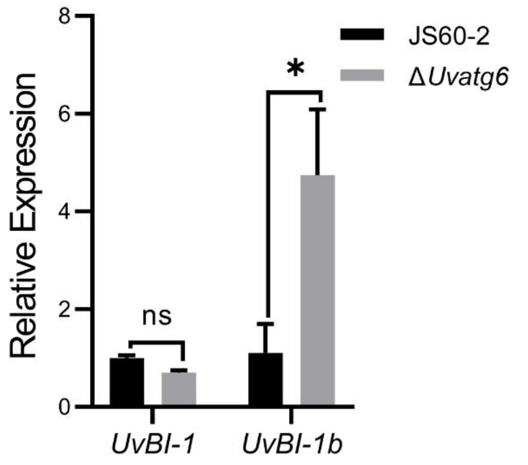

Fig. S10 Expression of *UvBI-1* and *UvBI-1b* in Δ*Uvatg6* transformants. The expression levels in the wild-type strain JS60-2 were normalized to one. The experiments were performed in three independent biological replicates with three technical replicates each time. Error bars represent standard deviation, and asterisks represent significant differences ($P < 0.05$).

Table S1 *U. virens* strains used in this study

	UvATG6	*UvBI-1b*	*UvBI-1/UvBI-1b*
Wild type	JS60-2	JZ11-28	JZ11-28
Knockout mutants	Δ*Uvatg6*-1	Δ*Uvbi-1b*-5	Δ*Uvbi-1/bi-1b*-2
	Δ*Uvatg6*-37	Δ*Uvbi-1b*-7	Δ*Uvbi-1/bi-1b*-5
Complemented mutants	*Uvatg6*-C	*Uvbi-1b*-C	

Table S2 Primers used in this study

Primer name	Primer sequence (5’-3’)
PVXBI-1bClaIF	CAGCACCAGCTAGCATCGATATGTCCTTCACCGTCAGCGT
PVXBI-1bSmaIR	AGTCCATGGATCCCCCGGGCTTGCGGCGGTTCTGCTGCA
BI-1b-CRISPR-F	ACCTGGACAAGTGAGTCAAAACCC
BI-1b-CRISPR-R	AAACGGGTTTTGACTCACTTGTCC
BI-1b-5F	CACAGCCGACATTTACGTGC
BI-1b-5R(HYG)	TCAATATCATCTTCTGTCGAGGTCGTCGAGAAGGTTGGGAAAC
BI-1b-3F(HYG)	AGATGCCGGATCCACTTAACCGAGGCGGGTAGGGAAGAAG
BI-1b-3R	GACACGCACATGCATCAAATCC
BI-1b-5N	GCTGGACCTGTCATATTGCAAG
BI-1b-3N-2	GCTGCATTGCGTCATTCGGTTC
pCETNS4_BI-1b(exon)_F	CAAACACAACCTTCAAAGAGCTCATGTCCTTCACCGTCAG
pCETNS4_BI-1b(exon)_R	CCATTTGTCTCAACCCCGGGTTACTTGCGGCGGTTCTGC
G418-F	GTTCTTTTTGTCAAGACCGACCTG
G418_R	CGATACCGTAAAGCACGAGGAAG
RT-BI-1b-F	CCGACAGCTTCTTCAGCTC
RT-BI-1b-R	GTTGAAGACGGCGTTGAT
CRISPR-UvATG6-F	ACCTACGTTCAAAAGACACCATGG
CRISPR-UvATG6-R	AAACCCATGGTGTCTTTTGAACGT
UvATG6-HYG-3F	AGATGCCGGATCCACTTAACGGCAGCTCAACGCGACAAAA
UvATG6-HYG-5R	TCAATATCATCTTCTGTCGAGTTCTGACGAGCTGTCTCTAG
UvATG6-3N	TGTGCGAGATTTTGCGGGAT
UvATG6-5N	CCCATGTGGTGTGGTTGAAA
UvATG6-3R	ACAAAATCCCGTCCAAACGG
UvATG6-5F	AATAACGTCCAGAGCGTCCG
H2	GGTAAATAGCTGCGCCGATGGTT
H3	GAACATCGCCTCGCTCCAGTCA
HYG-F	TCGACAGAAGATGATATTGAAGGAG
HYG-R	GTTAAGTGGATCCGGCATCT
UvATG6-F	ATGTTCTGCCAAAAGTGCAG
UvATG6-R	TCACGTCATTGTATTTCTAGCCT
UvATG6-3R-3N	GTCGTGGAAGCTTTTGTCGA
UvATG6-5F-5N	TTCGATGCCATCTTGGTCAC
Uv-tubulin-F	GGCGTTTACAATGGCACTTC
Uv-tubulin-R	CGGAACAGTTGACCAAAAGG
pNEO-F	TAAGTGAGTTACTGCGCACA
pNEO-R	AGATGAACTTCAGGGTCAGC
pNEO-UvATG6-F	TCGACGGTATCGATAAGCTTCCAGCTGGGCTCTTAAGGAG
pNEO-UvATG6-R	ATTCTAGAACTAGTGGATCCCGTCATTGTATTTCTAGCCTTTGA
UvATG6-RT-F	CTTCGGCTAGGGCGATTGT
UvATG6-RT-R	CGACTTGACGAGGGACTGG

UvATG8-RT-F	AGCAAATTCAAGGACGAGC
UvATG8-RT-R	GTCAAATCTGACGGCACCA
UvATG8-RT-F	AGCAAATTCAAGGACGAGC
UvATG8-RT-R	GTCAAATCTGACGGCACCA
UvBI-1b-RT-F	CCGACAGCTTCTTCAGCTC
UvBI-1b-RT-R	GTTGAAGACGGCGTTGAT
UvBI-1-RT-F	CTTCTACCAGACAGCTGTTG
UvBI-1-RT-R	GGAAGAACATGGCCATGAAG
UvATG12-RT-F	TCATGCTCGCCGACCTACC
UvATG12-RT-R	GCTTCCTCCGCAGATACCG
UvATG22-RT-F	GCATCCTGCATCACCACTT
UvATG22-RT-R	AGAATGCCGATGGACAGAC
UvATG9-RT-F	GGCGTGTTCGGAGGCATAT
UvATG9-RT-R	TGACGACCCGCATCTTGTA
GFP-RT-F	CAAGCTGACCCTGAAGTTC
GFP-RT-R	GTCGTCCTTGAAGAAGATG
NbAct-RT-F	GTTGCTATACAAGCTGTTCTCTCG
NbAct-RT-R	GTCAAGACGAAGAATGACATGTGG
Bax-RT-F	AGGATGCGTCCACCAAGAA
Bax-RT-R	AAGTAGAAGAGGGCAACCAC
PVX-UvATG6-F	CAGCACCAGCTAGCATCGATATGTTCTGCCAAAAGTGCAG
PVX-UvATG6-R	TAGTCCATGGATCCCCCGGGCGTCATTGTATTTCTAGCCTTTGAA
PVX-F	CACTCTCCGTTGAACGGTTA
PVX-R	GACCCTATGGGCTGTGTTG
BD-UvATG6-F	CATATGGCCATGGAGGCCATGTTCTGCCAAAAGTGCAG
BD-UvATG6-R	AGGTCGACGGATCCCCGGTCACGTCATTGTATTTCTAGCCTTT
T7-F	GACAGTTGACTGTATCGCCG
T7-R	GAAATTCGCCCGGAATTAGC
AD-UvBI-1-F	ATATGGCCATGGAGGCCAGTATGCAGCTCGTCATGCG
AD-UvBI-1-R	TCTGCAGCTCGAGCTCGATGTTGTTAGACTGGCTGTTGAGG
AD-F	GATACCCCACCAAACCCAAA
AD-R	GTGCACGATGCACAGTTGAA
AD-UvBI-1b-F	ATATGGCCATGGAGGCCAGTATGATCCTGCACCACGC
AD-UvBI-1b-R	ATCTGCAGCTCGAGCTCGATCTTGCGGCGGTTCTGC
4T-2-F	GGGCTGGCAAGCCACGTTTGGTG
4T-2-R	CCGGGAGCTGCATGTGTCAGAGG
4T-2-UvATG6-F	CGCGTGGATCCCCAGGAATTATGTTCTGCCAAAAGTGCAG
4T-2-UvATG6-R	GATGCGGCCGCTCGAGTCGACGTCATTGTATTTCTAGCCTTTGAA
32a-F	CGGTTCTGGTTCTGGCCATA
32a-R	TGCTAGTTATTGCTCAGCGG
32a-UvBI-1-F	CCGAATTCGAGCTCCGTCGAATGCAGCTCGTCATGCG

32a-UvBI-1-R	GGTGCTCGAGTGCGGCCGCATTAGTTGTTAGACTGGCTGTTGAG
32a-UvBI-1b-F	CCGAATTCGAGCTCCGTCGAATGATCCTGCACCACGC
32a-UvBI-1b-R	GGTGCTCGAGTGCGGCCGCATTACTTGCGGCGGTTCTGC
UvATG6-nLUC-F	CACAGTTCGAGAAGCTCGAGATGTTCTGCCAAAAGTGCAG
UvATG6-nLUC-R	CGGGACGCGTACGAGATCTGCGTCATTGTATTTCTAGCCTTTG
nLUC-F	CATTTCATTTGGAGAGAACACGG
nLUC-R	CATAGCCTTATGCAGTTGCTC
cLUC-UvBI-1-F	TCTCGTACGCGTCCCGGGGCATGGCCTCGAGCACCAC
cLUC-UvBI-1-R	TAGTCCATTTGTTGGATCCCGTTGTTAGACTGGCTGTTGAG
cLUC-F	TCTTACCGGAAAACTCGACG
cLUC-R	CTTGACGAACGTTGTCGAAA
cLUC-UvBI-1b-F	TCTCGTACGCGTCCCGGGGCATGTCCTTCACCGTCAGCG
cLUC-UvBI-1b-R	TAGTCCATTTGTTGGATCCCCTTGCGGCGGTTCTGCT
GFP-UvATG8-F	GCATGGACGAGCTGTACAAGATGCGAAGCAAATTCAAGGAC
GFP-UvATG8-R	GTCCTTGAATTTGCTTCGCATCTTGTACAGCTCGTCCATGC
p823-gfp-atg8-F	ATGGAGAAACTCGAGAATTCATCGCTGACTACATATCGCGTTC
p823-gfp-atg8-R	CAGTAACGTTAAGTGGATCCTTAGCCCTCTTGGAAAAAACCA
p823-CR	GCAGATGAGCTGTATCTGGAAG
p823-CF	GGGGATCTGGATTTTAGTACTGG
